# Thirteen moth species (Lepidoptera, Erebidae, Noctuidae) newly recorded in South Africa, with comments on their distribution

**DOI:** 10.3897/BDJ.10.e89729

**Published:** 2022-08-25

**Authors:** Sylvain Delabye, Fernando P Gaona, Pavel Potocký, Llewellyn C Foxcroft, Pavla Halamová, Martin Hejda, Sandra MacFadyen, Klára Pyšková, Ondřej Sedláček, Markéta Staňková, David Storch, Petr Pyšek, Robert Tropek

**Affiliations:** 1 Department of Ecology, Faculty of Science, Charles University, Prague, Czech Republic Department of Ecology, Faculty of Science, Charles University Prague Czech Republic; 2 Institute of Entomology, Biology Centre, Czech Academy of Sciences, Ceske Budejovice, Czech Republic Institute of Entomology, Biology Centre, Czech Academy of Sciences Ceske Budejovice Czech Republic; 3 Scientific Services, South African National Parks, Skukuza, South Africa Scientific Services, South African National Parks Skukuza South Africa; 4 Centre for Invasion Biology, Department of Botany and Zoology, Stellenbosch University, Stellenbosch, South Africa Centre for Invasion Biology, Department of Botany and Zoology, Stellenbosch University Stellenbosch South Africa; 5 Czech Academy of Sciences, Institute of Botany, Department of Invasion Ecology, Pruhonice, Czech Republic Czech Academy of Sciences, Institute of Botany, Department of Invasion Ecology Pruhonice Czech Republic; 6 Department of Mathematical Sciences, Stellenbosch University, Stellenbosch, South Africa Department of Mathematical Sciences, Stellenbosch University Stellenbosch South Africa; 7 Department of Zoology, Faculty of Science, Charles University, Prague, Czech Republic Department of Zoology, Faculty of Science, Charles University Prague Czech Republic; 8 Center for Theoretical Study, Charles University, Prague, Czech Republic Center for Theoretical Study, Charles University Prague Czech Republic

**Keywords:** Afrotropics, faunistic report, Heterocera, Kruger National Park, light trapping, savannahs, Zambezian region

## Abstract

**Background:**

Thanks to the high diversity of ecosystems and habitats, South Africa harbours tremendous diversity of insects. The Kruger National Park, due to its position close to the border between two biogeographic regions and high heterogeneity of environmental conditions, represents an insufficiently studied hotspot of lepidopteran diversity. During our ecological research in the Kruger National Park, we collected abundant moth material, including several interesting faunistic records reported in this study.

**New information:**

We reported 13 species of moths which had not yet been recorded in South Africa. In many cases, our records represented an important extension of the species’ known distribution, including two species (*Ozarbagaedei* and *O.persinua*) whose distribution ranges extended into the Zambezian biogeographic region. Such findings confirmed the poor regional knowledge of lepidopteran diversity.

## Introduction

South Africa offers an extraordinarily heterogeneous land area, hosting nine terrestrial biomes, including deserts, rich Mediterranean vegetation and various savannahs and forests (Mucina and Rutherford 2006). Located in the country’s north-eastern corner, the Kruger National Park (KNP) represents one of the largest African protected areas and one of the oldest national parks in the continent (Joubert 1986). It covers unusually heterogeneous savannah habitats from subtropical and tropical zones in its southern and northern parts, respectively. The 11 described land systems (defined by a combination of climate, geological conditions, altitude and vegetation character; Venter et al. 2003) belong to the Zambezian region, although some of them closely border with the Southern African region (Linder et al. 2012).

KNP hosts tremendous diversity of many taxonomic groups, including insects (Braack and Kryger 2003, Mawdsley et al. 2016, D'Souza et al. 2021). Even though KNP belongs to one of the South African areas with the greatest amount of moth distribution records (Mecenero et al. 2020), the knowledge on the Lepidoptera diversity still remains strongly insufficient. Our recent sampling of moths in a range of savannah habitats of KNP yielded more than 60,000 captured individuals. Amongst these, we identified 13 moth species recorded for the first time in South Africa. Here, we present the sampled material for these species, with remarks on their distribution.

## Materials and methods

All reported moth specimens were collected in the Kruger National Park, South Africa from November 2018 until March 2020, as a part of the MOSAIK project (Monitoring Savannah Biodiversity in the Kruger National Park) focused on exploring biodiversity patterns of plants and animals in various environmental settings of the savannah ecosystem (Pyšek et al. 2020, Hejda et al. 2022). Our sampling was performed at 60 plots at four main land systems (Skukuza, Satara, Phalaborwa and Letaba) between the Crocodile River in the south up to Punda Maria in the north (see Hejda et al. (2022) for the location of all plots). The plots were set up to cover three habitats with different levels of water availability and large herbivore disturbances (perennial rivers, seasonal rivers and crests with no availability of water; Fig. 1). Each plot was sampled during early (November) and late (February-March) wet seasons.

Moths were attracted and collected by portable light traps consisting of a two-sided strip of 48 LEDs emitting UV light and powered by 12V batteries (Delabye et al. 2020). Attracted specimens were anaesthetised by ammonium hydrogen carbonate inside the traps. In the collected moth material, we focused on twelve moth families: Erebidae, Eutellidae, Noctuidae, Nolidae, Notodontidae, Eupterotidae, Lasiocampidae, Saturniidae, Sphingidae, Geometridae, Thyrididae and Limacodidae. We identified all specimens of the focal families, based mostly on external morphology and/or genitalia dissections, using various available literature and online resources and the exhaustive Lepidoptera collection in the Nature Education Centre, Jagiellonian University, Kraków, Poland. The identification of all species reported here as the new country records were always confirmed by genitalia dissection, the reference literature being referred in comments to particular species.

The known distribution of individual species followed a combination of Hacker (2016), Hacker (2019) and the AfroMoths database (De Prins and De Prins 2022), with cross-checking various other available resources, including the Global Biodiversity Information Facility (GBIF; Robertson et al. 2014), the LepiMAP project (LepiMAP; Edge and Mecenero 2015), the iNaturalist database (iNaturalist; Nugent 2018) and the Barcoding of Life Data System (BOLD; Ratnasingham and Hebert 2007). The nomeclature of higher taxonomic units of Erebidae followed Zahiri et al. (2011). The voucher material was shared between collections of the Nature Education Centre of Jagiellonian University in Kraków, Poland and the Biology Centre, Czech Academy of Sciences, České Budějovice, Czechia.

## Taxon treatments

### Eublemma accedens aethiopica

Hacker, 2019

527BFC6C-7FC8-573C-8376-40FCE69F0ED8

Erebidae , Boletobiinae

#### Materials

- **Type status:** Other material. **Occurrence:** individualCount: 11; lifeStage: adult; **Taxon:** scientificName: Eublemmaaccedensaethiopica (Hacker, 2019); genus: Eublemma; specificEpithet: accedens aethiopica; scientificNameAuthorship: Hacker, 2019; **Location:** country: South Africa; stateProvince: Mpumalanga; verbatimLocality: Kruger NP, road S114, 3 km N of Historical Site 20; verbatimElevation: 366 m; decimalLatitude: -25.070953; decimalLongitude: 31.59927; **Identification:** identifiedBy: Fernando P. Gaona, Sylvain Delabye; dateIdentified: 2022; **Event:** eventDate: 02/27/2019; habitat: Crest; mixed thorn and Marula woodland savanna on granite: Bushwillow, Acacia and Marula; **Record Level:** type: PhysicalObject; institutionCode: IECA; basisOfRecord: PreservedSpecimen
- **Type status:** Other material. **Occurrence:** individualCount: 1; lifeStage: adult; **Taxon:** scientificName: Eublemmaaccedensaethiopica (Hacker, 2019); genus: Eublemma; specificEpithet: accedens aethiopica; scientificNameAuthorship: Hacker, 2019; **Location:** country: South Africa; stateProvince: Mpumalanga; verbatimLocality: Kruger NP, N’waswitshaka river, 4 km E of N’waswitshaka waterhole; verbatimElevation: 287 m; decimalLatitude: -25.026541; decimalLongitude: 31.54934; **Identification:** identifiedBy: Fernando P. Gaona, Sylvain Delabye; dateIdentified: 2022; **Event:** eventDate: 02/27/2019; habitat: Seasonal river; mixed woodland savanna and thorn thickets: Tamboti, Acacia, Marula and Bushwillows; **Record Level:** type: PhysicalObject; institutionCode: IECA; basisOfRecord: PreservedSpecimen
- **Type status:** Other material. **Occurrence:** individualCount: 2; lifeStage: adult; **Taxon:** scientificName: Eublemmaaccedensaethiopica (Hacker, 2019); genus: Eublemma; specificEpithet: accedens aethiopica; scientificNameAuthorship: Hacker, 2019; **Location:** country: South Africa; stateProvince: Mpumalanga; verbatimLocality: Kruger NP, Sabie river, road H4-1, 7.5 km E fo Skukuza camp; verbatimElevation: 265 m; decimalLatitude: -24.973743; decimalLongitude: 31.661434; **Identification:** identifiedBy: Fernando P. Gaona, Sylvain Delabye; dateIdentified: 2022; **Event:** eventDate: 02/27/2019; habitat: Perennial river; mixed woodland savanna and thorn thickets: Tamboti, Acacia, Marula and Bushwillow; **Record Level:** type: PhysicalObject; institutionCode: IECA; basisOfRecord: PreservedSpecimen
- **Type status:** Other material. **Occurrence:** individualCount: 7; lifeStage: adult; **Taxon:** scientificName: Eublemmaaccedensaethiopica (Hacker, 2019); genus: Eublemma; specificEpithet: accedens aethiopica; scientificNameAuthorship: Hacker, 2019; **Location:** country: South Africa; stateProvince: Mpumalanga; verbatimLocality: Kruger NP, road S26; verbatimElevation: 367 m; decimalLatitude: -25.208377; decimalLongitude: 31.652466; **Identification:** identifiedBy: Fernando P. Gaona, Sylvain Delabye; dateIdentified: 2022; **Event:** eventDate: 02/24/2019; habitat: Crest; mixed thorn and Marula woodland savanna on granite: Bushwillow, Acacia and Marula; **Record Level:** type: PhysicalObject; institutionCode: IECA; basisOfRecord: PreservedSpecimen
- **Type status:** Other material. **Occurrence:** individualCount: 86; lifeStage: adult; **Taxon:** scientificName: Eublemmaaccedensaethiopica (Hacker, 2019); genus: Eublemma; specificEpithet: accedens aethiopica; scientificNameAuthorship: Hacker, 2019; **Location:** country: South Africa; stateProvince: Mpumalanga; verbatimLocality: Kruger NP, Sabie river, Lookout on S79 road; verbatimElevation: 200 m; decimalLatitude: -25.064099; decimalLongitude: 31.825735; **Identification:** identifiedBy: Fernando P. Gaona, Sylvain Delabye; dateIdentified: 2022; **Event:** eventDate: 02/24/2019; habitat: Perennial river; mixed woodland savanna and thorn thickets: Tamboti, Acacia, Marula and Bushwillow; **Record Level:** type: PhysicalObject; institutionCode: IECA; basisOfRecord: PreservedSpecimen
- **Type status:** Other material. **Occurrence:** individualCount: 2; lifeStage: adult; **Taxon:** scientificName: Eublemmaaccedensaethiopica (Hacker, 2019); genus: Eublemma; specificEpithet: accedens aethiopica; scientificNameAuthorship: Hacker, 2019; **Location:** country: South Africa; stateProvince: Mpumalanga; verbatimLocality: Kruger NP, Crocodile river, road S114, 5 km N of Malelane gate; verbatimElevation: 300 m; decimalLatitude: -25.437282; decimalLongitude: 31.528655; **Identification:** identifiedBy: Fernando P. Gaona, Sylvain Delabye; dateIdentified: 2022; **Event:** eventDate: 02/25/2019; habitat: Perennial river; mixed woodland savanna and thorn thickets: Tamboti, Acacia, Marula and Bushwillow; **Record Level:** type: PhysicalObject; institutionCode: IECA; basisOfRecord: PreservedSpecimen
- **Type status:** Other material. **Occurrence:** individualCount: 2; lifeStage: adult; **Taxon:** scientificName: Eublemmaaccedensaethiopica (Hacker, 2019); genus: Eublemma; specificEpithet: accedens aethiopica; scientificNameAuthorship: Hacker, 2019; **Location:** country: South Africa; stateProvince: Mpumalanga; verbatimLocality: Kruger NP, Jock waterhole; verbatimElevation: 381 m; decimalLatitude: -25.301012; decimalLongitude: 31.549768; **Identification:** identifiedBy: Fernando P. Gaona, Sylvain Delabye; dateIdentified: 2022; **Event:** eventDate: 02/25/2019; habitat: Crest; mixed woodland savanna and thorn thickets: Tamboti, Acacia, Marula and Bushwillow; **Record Level:** type: PhysicalObject; institutionCode: IECA; basisOfRecord: PreservedSpecimen
- **Type status:** Other material. **Occurrence:** individualCount: 2; lifeStage: adult; **Taxon:** scientificName: Eublemmaaccedensaethiopica (Hacker, 2019); genus: Eublemma; specificEpithet: accedens aethiopica; scientificNameAuthorship: Hacker, 2019; **Location:** country: South Africa; stateProvince: Mpumalanga; verbatimLocality: Kruger NP, Biyamiti river, road S23, 1 km N of Wehr dam; verbatimElevation: 290 m; decimalLatitude: -25.266171; decimalLongitude: 31.619022; **Identification:** identifiedBy: Fernando P. Gaona, Sylvain Delabye; dateIdentified: 2022; **Event:** eventDate: 11/07/2018; habitat: Seasonal river; mixed thorn and Marula woodland savanna on granite: Bushwillow, Acacia and Marula; **Record Level:** type: PhysicalObject; institutionCode: IECA; basisOfRecord: PreservedSpecimen
- **Type status:** Other material. **Occurrence:** individualCount: 2; lifeStage: adult; **Taxon:** scientificName: Eublemmaaccedensaethiopica (Hacker, 2019); genus: Eublemma; specificEpithet: accedens aethiopica; scientificNameAuthorship: Hacker, 2019; **Location:** country: South Africa; stateProvince: Mpumalanga; verbatimLocality: Kruger NP, road S36, 5 km NE of Hlanguleni picnic site; verbatimElevation: 333 m; decimalLatitude: -24.681967; decimalLongitude: 31.666664; **Identification:** identifiedBy: Fernando P. Gaona, Sylvain Delabye; dateIdentified: 2022; **Event:** eventDate: 03/03/2019; habitat: Crest; mixed thorn and Marula woodland savanna on granite: Bushwillow, Acacia and Marula; **Record Level:** type: PhysicalObject; institutionCode: IECA; basisOfRecord: PreservedSpecimen
- **Type status:** Other material. **Occurrence:** individualCount: 1; lifeStage: adult; **Taxon:** scientificName: Eublemmaaccedensaethiopica (Hacker, 2019); genus: Eublemma; specificEpithet: accedens aethiopica; scientificNameAuthorship: Hacker, 2019; **Location:** country: South Africa; stateProvince: Mpumalanga; verbatimLocality: Kruger NP, Gayisenga waterhole; verbatimElevation: 256 m; decimalLatitude: -25.270645; decimalLongitude: 31.756561; **Identification:** identifiedBy: Fernando P. Gaona, Sylvain Delabye; dateIdentified: 2022; **Event:** eventDate: 03/06/2019; habitat: Seasonal river; mixed thorn and Marula woodland savanna on granite: Bushwillow, Acacia and Marula; **Record Level:** type: PhysicalObject; institutionCode: IECA; basisOfRecord: PreservedSpecimen
- **Type status:** Other material. **Occurrence:** individualCount: 5; lifeStage: adult; **Taxon:** scientificName: Eublemmaaccedensaethiopica (Hacker, 2019); genus: Eublemma; specificEpithet: accedens aethiopica; scientificNameAuthorship: Hacker, 2019; **Location:** country: South Africa; stateProvince: Mpumalanga; verbatimLocality: Kruger NP, Ripape river, S33 and S36 crossroads; verbatimElevation: 325 m; decimalLatitude: -24.757101; decimalLongitude: 31.698319; **Identification:** identifiedBy: Fernando P. Gaona, Sylvain Delabye; dateIdentified: 2022; **Event:** eventDate: 03/03/2019; habitat: Seasonal river; mixed thorn and Marula woodland savanna on granite: Bushwillow, Acacia and Marula; **Record Level:** type: PhysicalObject; institutionCode: IECA; basisOfRecord: PreservedSpecimen
- **Type status:** Other material. **Occurrence:** individualCount: 14; lifeStage: adult; **Taxon:** scientificName: Eublemmaaccedensaethiopica (Hacker, 2019); genus: Eublemma; specificEpithet: accedens aethiopica; scientificNameAuthorship: Hacker, 2019; **Location:** country: South Africa; stateProvince: Mpumalanga; verbatimLocality: Kruger NP, N’watimhiri river, road S21, 1 km NE of Nhlotini waterhole; verbatimElevation: 283 m; decimalLatitude: -25.116682; decimalLongitude: 31.696896; **Identification:** identifiedBy: Fernando P. Gaona, Sylvain Delabye; dateIdentified: 2022; **Event:** eventDate: 02/24/2019; habitat: Seasonal river; mixed woodland savanna and thorn thickets: Tamboti, Acacia, Marula and Bushwillows; **Record Level:** type: PhysicalObject; institutionCode: IECA; basisOfRecord: PreservedSpecimen
- **Type status:** Other material. **Occurrence:** individualCount: 2; lifeStage: adult; **Taxon:** scientificName: Eublemmaaccedensaethiopica (Hacker, 2019); genus: Eublemma; specificEpithet: accedens aethiopica; scientificNameAuthorship: Hacker, 2019; **Location:** country: South Africa; stateProvince: Mpumalanga; verbatimLocality: Kruger NP, road H5; verbatimElevation: 307 m; decimalLatitude: -25.20729; decimalLongitude: 31.77072; **Identification:** identifiedBy: Fernando P. Gaona, Sylvain Delabye; dateIdentified: 2022; **Event:** eventDate: 03/06/2019; habitat: Crest; mixed thorn and Marula woodland savanna on granite: Bushwillow, Acacia and Marula; **Record Level:** type: PhysicalObject; institutionCode: IECA; basisOfRecord: PreservedSpecimen
- **Type status:** Other material. **Occurrence:** individualCount: 8; lifeStage: adult; **Taxon:** scientificName: Eublemmaaccedensaethiopica (Hacker, 2019); genus: Eublemma; specificEpithet: accedens aethiopica; scientificNameAuthorship: Hacker, 2019; **Location:** country: South Africa; stateProvince: Mpumalanga; verbatimLocality: Kruger NP, N’waswitsontso river, road S125, 1 km NW of Mhisanamond waterhole; verbatimElevation: 329 m; decimalLatitude: -24.600415; decimalLongitude: 31.687177; **Identification:** identifiedBy: Fernando P. Gaona, Sylvain Delabye; dateIdentified: 2022; **Event:** eventDate: 03/03/2019; habitat: Perennial river; mixed thorn and Marula woodland savanna on granite: Bushwillow, Acacia and Marula; **Record Level:** type: PhysicalObject; institutionCode: IECA; basisOfRecord: PreservedSpecimen
- **Type status:** Other material. **Occurrence:** individualCount: 1; lifeStage: adult; **Taxon:** scientificName: Eublemmaaccedensaethiopica (Hacker, 2019); genus: Eublemma; specificEpithet: accedens aethiopica; scientificNameAuthorship: Hacker, 2019; **Location:** country: South Africa; stateProvince: Mpumalanga; verbatimLocality: Kruger NP, road H10, 1 km SE of Nkumbe View Site; verbatimElevation: 407 m; decimalLatitude: -24.861873; decimalLongitude: 31.897264; **Identification:** identifiedBy: Fernando P. Gaona, Sylvain Delabye; dateIdentified: 2022; **Event:** eventDate: 02/26/2019; habitat: Crest; open savanna grassland on basalt; **Record Level:** type: PhysicalObject; institutionCode: IECA; basisOfRecord: PreservedSpecimen
- **Type status:** Other material. **Occurrence:** individualCount: 1; lifeStage: adult; **Taxon:** scientificName: Eublemmaaccedensaethiopica (Hacker, 2019); genus: Eublemma; specificEpithet: accedens aethiopica; scientificNameAuthorship: Hacker, 2019; **Location:** country: South Africa; stateProvince: Mpumalanga; verbatimLocality: Kruger NP, Orpen dam; verbatimElevation: 253 m; decimalLatitude: -24.793037; decimalLongitude: 31.899386; **Identification:** identifiedBy: Fernando P. Gaona, Sylvain Delabye; dateIdentified: 2022; **Event:** eventDate: 02/26/2019; habitat: Perennial river; Lebombo - low, arid rhyolite hills: Acacia and thorn thickets; **Record Level:** type: PhysicalObject; institutionCode: IECA; basisOfRecord: PreservedSpecimen
- **Type status:** Other material. **Occurrence:** individualCount: 1; lifeStage: adult; **Taxon:** scientificName: Eublemmaaccedensaethiopica (Hacker, 2019); genus: Eublemma; specificEpithet: accedens aethiopica; scientificNameAuthorship: Hacker, 2019; **Location:** country: South Africa; stateProvince: Mpumalanga; verbatimLocality: Kruger NP, Silolweni river, road H1-2, 1 km S of Silolweni dam; verbatimElevation: 284 m; decimalLatitude: -24.820865; decimalLongitude: 31.844014; **Identification:** identifiedBy: Fernando P. Gaona, Sylvain Delabye; dateIdentified: 2022; **Event:** eventDate: 02/26/2019; habitat: Seasonal river; open savanna grassland on basalt; **Record Level:** type: PhysicalObject; institutionCode: IECA; basisOfRecord: PreservedSpecimen
- **Type status:** Other material. **Occurrence:** individualCount: 1; lifeStage: adult; **Taxon:** scientificName: Eublemmaaccedensaethiopica (Hacker, 2019); genus: Eublemma; specificEpithet: accedens aethiopica; scientificNameAuthorship: Hacker, 2019; **Location:** country: South Africa; stateProvince: Mpumalanga; verbatimLocality: Kruger NP, Sweni Bird Hide; verbatimElevation: 187 m; decimalLatitude: -24.474196; decimalLongitude: 31.972276; **Identification:** identifiedBy: Fernando P. Gaona, Sylvain Delabye; dateIdentified: 2022; **Event:** eventDate: 03/02/2019; habitat: Perennial river; Oliphants rugged veld: mixed thornveld and woodland savanna; **Record Level:** type: PhysicalObject; institutionCode: IECA; basisOfRecord: PreservedSpecimen
- **Type status:** Other material. **Occurrence:** individualCount: 9; lifeStage: adult; **Taxon:** scientificName: Eublemmaaccedensaethiopica (Hacker, 2019); genus: Eublemma; specificEpithet: accedens aethiopica; scientificNameAuthorship: Hacker, 2019; **Location:** country: South Africa; stateProvince: Mpumalanga; verbatimLocality: Kruger NP, N’wanetzi river, road S100, 2 km W of Nsasane waterhole; verbatimElevation: 228 m; decimalLatitude: -24.381219; decimalLongitude: 31.886093; **Identification:** identifiedBy: Fernando P. Gaona, Sylvain Delabye; dateIdentified: 2022; **Event:** eventDate: 03/02/2019; habitat: Seasonal river; Oliphants rugged veld: mixed thornveld and woodland savanna; **Record Level:** type: PhysicalObject; institutionCode: IECA; basisOfRecord: PreservedSpecimen
- **Type status:** Other material. **Occurrence:** individualCount: 1; lifeStage: adult; **Taxon:** scientificName: Eublemmaaccedensaethiopica (Hacker, 2019); genus: Eublemma; specificEpithet: accedens aethiopica; scientificNameAuthorship: Hacker, 2019; **Location:** country: South Africa; stateProvince: Mpumalanga; verbatimLocality: Kruger NP, road H1-3, 500m W of Marheya waterhole; verbatimElevation: 333 m; decimalLatitude: -24.565596; decimalLongitude: 31.77476; **Identification:** identifiedBy: Fernando P. Gaona, Sylvain Delabye; dateIdentified: 2022; **Event:** eventDate: 03/01/2019; habitat: Crest; wooded savanna on shale: Bushwillow, Leadwood, Marula, Knobthorn trees, and thorn thickets; **Record Level:** type: PhysicalObject; institutionCode: IECA; basisOfRecord: PreservedSpecimen
- **Type status:** Other material. **Occurrence:** individualCount: 2; lifeStage: adult; **Taxon:** scientificName: Eublemmaaccedensaethiopica (Hacker, 2019); genus: Eublemma; specificEpithet: accedens aethiopica; scientificNameAuthorship: Hacker, 2019; **Location:** country: South Africa; stateProvince: Mpumalanga; verbatimLocality: Kruger NP, Crocodile river, Crocodile Bridge; verbatimElevation: 183 m; decimalLatitude: -25.363011; decimalLongitude: 31.891832; **Identification:** identifiedBy: Fernando P. Gaona, Sylvain Delabye; dateIdentified: 2022; **Event:** eventDate: 03/07/2019; habitat: Perennial river; open savanna grassland on basalt; **Record Level:** type: PhysicalObject; institutionCode: IECA; basisOfRecord: PreservedSpecimen
- **Type status:** Other material. **Occurrence:** individualCount: 1; lifeStage: adult; **Taxon:** scientificName: Eublemmaaccedensaethiopica (Hacker, 2019); genus: Eublemma; specificEpithet: accedens aethiopica; scientificNameAuthorship: Hacker, 2019; **Location:** country: South Africa; stateProvince: Mpumalanga; verbatimLocality: Kruger NP, road S36, 7 km S of Hamiltons Tented camp; verbatimElevation: 242 m; decimalLatitude: -25.283896; decimalLongitude: 31.926798; **Identification:** identifiedBy: Fernando P. Gaona, Sylvain Delabye; dateIdentified: 2022; **Event:** eventDate: 03/07/2019; habitat: Crest; open savanna grassland on basalt; **Record Level:** type: PhysicalObject; institutionCode: IECA; basisOfRecord: PreservedSpecimen
- **Type status:** Other material. **Occurrence:** individualCount: 3; lifeStage: adult; **Taxon:** scientificName: Eublemmaaccedensaethiopica (Hacker, 2019); genus: Eublemma; specificEpithet: accedens aethiopica; scientificNameAuthorship: Hacker, 2019; **Location:** country: South Africa; stateProvince: Limpopo; verbatimLocality: Kruger NP, Ngwenyeni river, 3 km NE of Nandzana waterhole; verbatimElevation: 335 m; decimalLatitude: -23.817311; decimalLongitude: 31.296011; **Identification:** identifiedBy: Fernando P. Gaona, Sylvain Delabye; dateIdentified: 2022; **Event:** eventDate: 02/19/2020; habitat: Seasonal river; mopani dominated woodland savanna on granite: Acacia, Bushwillow and Mopane; **Record Level:** type: PhysicalObject; institutionCode: IECA (x1); ZMJU (x2); basisOfRecord: PreservedSpecimen
- **Type status:** Other material. **Occurrence:** individualCount: 5; lifeStage: adult; **Taxon:** scientificName: Eublemmaaccedensaethiopica (Hacker, 2019); genus: Eublemma; specificEpithet: accedens aethiopica; scientificNameAuthorship: Hacker, 2019; **Location:** country: South Africa; stateProvince: Limpopo; verbatimLocality: Kruger NP, Letaba river, 5 km E of road H14; verbatimElevation: 278 m; decimalLatitude: -23.757038; decimalLongitude: 31.392944; **Identification:** identifiedBy: Fernando P. Gaona, Sylvain Delabye; dateIdentified: 2022; **Event:** eventDate: 02/19/2020; habitat: Perennial river; mopani dominated woodland savanna on granite: Acacia, Bushwillow and Mopane; **Record Level:** type: PhysicalObject; institutionCode: IECA; basisOfRecord: PreservedSpecimen
- **Type status:** Other material. **Occurrence:** individualCount: 2; lifeStage: adult; **Taxon:** scientificName: Eublemmaaccedensaethiopica (Hacker, 2019); genus: Eublemma; specificEpithet: accedens aethiopica; scientificNameAuthorship: Hacker, 2019; **Location:** country: South Africa; stateProvince: Limpopo; verbatimLocality: Kruger NP, road S133, 6 km S of Jumbo waterhole; verbatimElevation: 365 m; decimalLatitude: -23.844016; decimalLongitude: 31.365157; **Identification:** identifiedBy: Fernando P. Gaona, Sylvain Delabye; dateIdentified: 2022; **Event:** eventDate: 02/19/2020; habitat: Crest; mixed woodland savanna with sweet grazing: Knobthorn and Marula; **Record Level:** type: PhysicalObject; institutionCode: CB (x2); ZMJU (x1); basisOfRecord: PreservedSpecimen
- **Type status:** Other material. **Occurrence:** individualCount: 1; lifeStage: adult; **Taxon:** scientificName: Eublemmaaccedensaethiopica (Hacker, 2019); genus: Eublemma; specificEpithet: accedens aethiopica; scientificNameAuthorship: Hacker, 2019; **Location:** country: South Africa; stateProvince: Limpopo; verbatimLocality: Kruger NP, Nhlanganini dam; verbatimElevation: 327 m; decimalLatitude: -23.933918; decimalLongitude: 31.494673; **Identification:** identifiedBy: Fernando P. Gaona, Sylvain Delabye; dateIdentified: 2022; **Event:** eventDate: 02/21/2020; habitat: Perennial river; mixed woodland savanna with sweet grazing: Knobthorn and Marula; **Record Level:** type: PhysicalObject; institutionCode: IECA; basisOfRecord: PreservedSpecimen
- **Type status:** Other material. **Occurrence:** individualCount: 5; lifeStage: adult; **Taxon:** scientificName: Eublemmaaccedensaethiopica (Hacker, 2019); genus: Eublemma; specificEpithet: accedens aethiopica; scientificNameAuthorship: Hacker, 2019; **Location:** country: South Africa; stateProvince: Limpopo; verbatimLocality: Kruger NP, road H9, close to Misumani river; verbatimElevation: 379 m; decimalLatitude: -23.930505; decimalLongitude: 31.398723; **Identification:** identifiedBy: Fernando P. Gaona, Sylvain Delabye; dateIdentified: 2022; **Event:** eventDate: 02/21/2020; habitat: Crest; mixed woodland savanna with sweet grazing: Knobthorn and Marula; **Record Level:** type: PhysicalObject; institutionCode: IECA; basisOfRecord: PreservedSpecimen
- **Type status:** Other material. **Occurrence:** individualCount: 2; lifeStage: adult; **Taxon:** scientificName: Eublemmaaccedensaethiopica (Hacker, 2019); genus: Eublemma; specificEpithet: accedens aethiopica; scientificNameAuthorship: Hacker, 2019; **Location:** country: South Africa; stateProvince: Limpopo; verbatimLocality: Kruger NP, road S47, Nwanedzi river; verbatimElevation: 278 m; decimalLatitude: -23.793044; decimalLongitude: 31.47949; **Identification:** identifiedBy: Fernando P. Gaona, Sylvain Delabye; dateIdentified: 2022; **Event:** eventDate: 02/21/2020; habitat: Seasonal river; mopani dominated woodland savanna on granite: Acacia, Bushwillow and Mopane; **Record Level:** type: PhysicalObject; institutionCode: IECA; basisOfRecord: PreservedSpecimen
- **Type status:** Other material. **Occurrence:** individualCount: 1; lifeStage: adult; **Taxon:** scientificName: Eublemmaaccedensaethiopica (Hacker, 2019); genus: Eublemma; specificEpithet: accedens aethiopica; scientificNameAuthorship: Hacker, 2019; **Location:** country: South Africa; stateProvince: Limpopo; verbatimLocality: Kruger NP, road S47, Nwanedzi river; verbatimElevation: 278 m; decimalLatitude: -23.793044; decimalLongitude: 31.47949; **Identification:** identifiedBy: Fernando P. Gaona, Sylvain Delabye; dateIdentified: 2022; **Event:** eventDate: 12/02/2019; habitat: Seasonal river; mopani dominated woodland savanna on granite: Acacia, Bushwillow and Mopane; **Record Level:** type: PhysicalObject; institutionCode: IECA; basisOfRecord: PreservedSpecimen
- **Type status:** Other material. **Occurrence:** individualCount: 1; lifeStage: adult; **Taxon:** scientificName: Eublemmaaccedensaethiopica (Hacker, 2019); genus: Eublemma; specificEpithet: accedens aethiopica; scientificNameAuthorship: Hacker, 2019; **Location:** country: South Africa; stateProvince: Limpopo; verbatimLocality: Kruger NP, Stapelkop dam; verbatimElevation: 362 m; decimalLatitude: -23.594645; decimalLongitude: 31.254819; **Identification:** identifiedBy: Fernando P. Gaona, Sylvain Delabye; dateIdentified: 2022; **Event:** eventDate: 11/30/2019; habitat: Perennial river; mopani dominated woodland savanna on granite: Acacia, Bushwillow and Mopane; **Record Level:** type: PhysicalObject; institutionCode: IECA; basisOfRecord: PreservedSpecimen
- **Type status:** Other material. **Occurrence:** individualCount: 4; lifeStage: adult; **Taxon:** scientificName: Eublemmaaccedensaethiopica (Hacker, 2019); genus: Eublemma; specificEpithet: accedens aethiopica; scientificNameAuthorship: Hacker, 2019; **Location:** country: South Africa; stateProvince: Limpopo; verbatimLocality: Kruger NP, Tsendze river, 2 km SE of Frazerus waterhole; verbatimElevation: 352 m; decimalLatitude: -23.495737; decimalLongitude: 31.325834; **Identification:** identifiedBy: Fernando P. Gaona, Sylvain Delabye; dateIdentified: 2022; **Event:** eventDate: 02/28/2020; habitat: Seasonal river; mopani dominated woodland savanna on granite: Acacia, Bushwillow and Mopane; **Record Level:** type: PhysicalObject; institutionCode: ZMJU; basisOfRecord: PreservedSpecimen
- **Type status:** Other material. **Occurrence:** individualCount: 2; lifeStage: adult; **Taxon:** scientificName: Eublemmaaccedensaethiopica (Hacker, 2019); genus: Eublemma; specificEpithet: accedens aethiopica; scientificNameAuthorship: Hacker, 2019; **Location:** country: South Africa; stateProvince: Limpopo; verbatimLocality: Kruger NP, road H1-6, 1 km SW of Olifantsbad pan; verbatimElevation: 426 m; decimalLatitude: -23.335829; decimalLongitude: 31.32542; **Identification:** identifiedBy: Fernando P. Gaona, Sylvain Delabye; dateIdentified: 2022; **Event:** eventDate: 02/24/2020; habitat: Crest; mopani dominated woodland savanna on granite: Acacia, Bushwillow and Mopane; **Record Level:** type: PhysicalObject; institutionCode: CB (x1); ZMJU (x1); basisOfRecord: PreservedSpecimen
- **Type status:** Other material. **Occurrence:** individualCount: 3; lifeStage: adult; **Taxon:** scientificName: Eublemmaaccedensaethiopica (Hacker, 2019); genus: Eublemma; specificEpithet: accedens aethiopica; scientificNameAuthorship: Hacker, 2019; **Location:** country: South Africa; stateProvince: Limpopo; verbatimLocality: Kruger NP, Redrocks waterhole; verbatimElevation: 328 m; decimalLatitude: -23.172717; decimalLongitude: 31.299895; **Identification:** identifiedBy: Fernando P. Gaona, Sylvain Delabye; dateIdentified: 2022; **Event:** eventDate: 02/24/2020; habitat: Perennial river; open savanna grassland with stunted mopane; **Record Level:** type: PhysicalObject; institutionCode: IECA; basisOfRecord: PreservedSpecimen
- **Type status:** Other material. **Occurrence:** individualCount: 1; lifeStage: adult; **Taxon:** scientificName: Eublemmaaccedensaethiopica (Hacker, 2019); genus: Eublemma; specificEpithet: accedens aethiopica; scientificNameAuthorship: Hacker, 2019; **Location:** country: South Africa; stateProvince: Limpopo; verbatimLocality: Kruger NP, Shisha West waterhole; verbatimElevation: 363 m; decimalLatitude: -22.8274; decimalLongitude: 31.216057; **Identification:** identifiedBy: Fernando P. Gaona, Sylvain Delabye; dateIdentified: 2022; **Event:** eventDate: 02/25/2020; habitat: Seasonal river; open savanna grassland with stunted mopane; **Record Level:** type: PhysicalObject; institutionCode: IECA; basisOfRecord: PreservedSpecimen
- **Type status:** Other material. **Occurrence:** individualCount: 2; lifeStage: adult; **Taxon:** scientificName: Eublemmaaccedensaethiopica (Hacker, 2019); genus: Eublemma; specificEpithet: accedens aethiopica; scientificNameAuthorship: Hacker, 2019; **Location:** country: South Africa; stateProvince: Limpopo; verbatimLocality: Kruger NP, management track at Babalala; verbatimElevation: 357 m; decimalLatitude: -22.898804; decimalLongitude: 31.284004; **Identification:** identifiedBy: Fernando P. Gaona, Sylvain Delabye; dateIdentified: 2022; **Event:** eventDate: 02/25/2020; habitat: Crest; open savanna grassland with stunted mopane; **Record Level:** type: PhysicalObject; institutionCode: CB (x1); ZMJU (x1); basisOfRecord: PreservedSpecimen
- **Type status:** Other material. **Occurrence:** individualCount: 2; lifeStage: adult; **Taxon:** scientificName: Eublemmaaccedensaethiopica (Hacker, 2019); genus: Eublemma; specificEpithet: accedens aethiopica; scientificNameAuthorship: Hacker, 2019; **Location:** country: South Africa; stateProvince: Limpopo; verbatimLocality: Kruger NP, road S56, Mphongolo river, 1 km N of Ribye waterhole; verbatimElevation: 314 m; decimalLatitude: -22.991653; decimalLongitude: 31.270644; **Identification:** identifiedBy: Fernando P. Gaona, Sylvain Delabye; dateIdentified: 2022; **Event:** eventDate: 02/25/2020; habitat: Perennial river; open savanna grassland with stunted mopane; **Record Level:** type: PhysicalObject; institutionCode: ZMJU; basisOfRecord: PreservedSpecimen
- **Type status:** Other material. **Occurrence:** individualCount: 4; lifeStage: adult; **Taxon:** scientificName: Eublemmaaccedensaethiopica (Hacker, 2019); genus: Eublemma; specificEpithet: accedens aethiopica; scientificNameAuthorship: Hacker, 2019; **Location:** country: South Africa; stateProvince: Mpumalanga; verbatimLocality: Kruger NP, Timbavati river, road S39, 6 km N of Roodewal camp; verbatimElevation: 249 m; decimalLatitude: -24.10563; decimalLongitude: 31.660381; **Identification:** identifiedBy: Fernando P. Gaona, Sylvain Delabye; dateIdentified: 2022; **Event:** eventDate: 02/17/2020; habitat: Seasonal river; open savanna grassland with stunted mopane; **Record Level:** type: PhysicalObject; institutionCode: IECA; basisOfRecord: PreservedSpecimen
- **Type status:** Other material. **Occurrence:** individualCount: 2; lifeStage: adult; **Taxon:** scientificName: Eublemmaaccedensaethiopica (Hacker, 2019); genus: Eublemma; specificEpithet: accedens aethiopica; scientificNameAuthorship: Hacker, 2019; **Location:** country: South Africa; stateProvince: Mpumalanga; verbatimLocality: Kruger NP, S90 (Old Main Road), 5 km SE of Balule camp; verbatimElevation: 288 m; decimalLatitude: -24.085694; decimalLongitude: 31.780033; **Identification:** identifiedBy: Fernando P. Gaona, Sylvain Delabye; dateIdentified: 2022; **Event:** eventDate: 02/17/2020; habitat: Crest; Oliphants rugged veld: mixed thornveld and woodland savanna; **Record Level:** type: PhysicalObject; institutionCode: IECA; basisOfRecord: PreservedSpecimen
- **Type status:** Other material. **Occurrence:** individualCount: 1; lifeStage: adult; **Taxon:** scientificName: Eublemmaaccedensaethiopica (Hacker, 2019); genus: Eublemma; specificEpithet: accedens aethiopica; scientificNameAuthorship: Hacker, 2019; **Location:** country: South Africa; stateProvince: Limpopo; verbatimLocality: Kruger NP, Makhadzi waterhole; verbatimElevation: 285 m; decimalLatitude: -23.697166; decimalLongitude: 31.617761; **Identification:** identifiedBy: Fernando P. Gaona, Sylvain Delabye; dateIdentified: 2022; **Event:** eventDate: 12/01/2019; habitat: Seasonal river; open savanna grassland with stunted mopane; **Record Level:** type: PhysicalObject; institutionCode: IECA; basisOfRecord: PreservedSpecimen
- **Type status:** Other material. **Occurrence:** individualCount: 1; lifeStage: adult; **Taxon:** scientificName: Eublemmaaccedensaethiopica (Hacker, 2019); genus: Eublemma; specificEpithet: accedens aethiopica; scientificNameAuthorship: Hacker, 2019; **Location:** country: South Africa; stateProvince: Limpopo; verbatimLocality: Kruger NP, road H1-5, 11 km S of Letaba camp; verbatimElevation: 301 m; decimalLatitude: -23.930371; decimalLongitude: 31.600825; **Identification:** identifiedBy: Fernando P. Gaona, Sylvain Delabye; dateIdentified: 2022; **Event:** eventDate: 02/20/2020; habitat: Crest; open savanna grassland with stunted mopane; **Record Level:** type: PhysicalObject; institutionCode: IECA; basisOfRecord: PreservedSpecimen
- **Type status:** Other material. **Occurrence:** individualCount: 1; lifeStage: adult; **Taxon:** scientificName: Eublemmaaccedensaethiopica (Hacker, 2019); genus: Eublemma; specificEpithet: accedens aethiopica; scientificNameAuthorship: Hacker, 2019; **Location:** country: South Africa; stateProvince: Limpopo; verbatimLocality: Kruger NP, Letaba river, 10 km E of Letaba camp; verbatimElevation: 217 m; decimalLatitude: -23.844905; decimalLongitude: 31.641514; **Identification:** identifiedBy: Fernando P. Gaona, Sylvain Delabye; dateIdentified: 2022; **Event:** eventDate: 02/20/2020; habitat: Perennial river; open savanna grassland with stunted mopane; **Record Level:** type: PhysicalObject; institutionCode: IECA; basisOfRecord: PreservedSpecimen
- **Type status:** Other material. **Occurrence:** individualCount: 1; lifeStage: adult; **Taxon:** scientificName: Eublemmaaccedensaethiopica (Hacker, 2019); genus: Eublemma; specificEpithet: accedens aethiopica; scientificNameAuthorship: Hacker, 2019; **Location:** country: South Africa; stateProvince: Limpopo; verbatimLocality: Kruger NP, Makhadzi waterhole; verbatimElevation: 285 m; decimalLatitude: -23.697166; decimalLongitude: 31.617761; **Identification:** identifiedBy: Fernando P. Gaona, Sylvain Delabye; dateIdentified: 2022; **Event:** eventDate: 02/20/2020; habitat: Seasonal river; open savanna grassland with stunted mopane; **Record Level:** type: PhysicalObject; institutionCode: IECA; basisOfRecord: PreservedSpecimen
- **Type status:** Other material. **Occurrence:** individualCount: 1; lifeStage: adult; **Taxon:** scientificName: Eublemmaaccedensaethiopica (Hacker, 2019); genus: Eublemma; specificEpithet: accedens aethiopica; scientificNameAuthorship: Hacker, 2019; **Location:** country: South Africa; stateProvince: Limpopo; verbatimLocality: Kruger NP, Middelvlei waterhole; verbatimElevation: 297 m; decimalLatitude: -23.68666; decimalLongitude: 31.546334; **Identification:** identifiedBy: Fernando P. Gaona, Sylvain Delabye; dateIdentified: 2022; **Event:** eventDate: 02/18/2020; habitat: Crest; open savanna grassland with stunted mopane; **Record Level:** type: PhysicalObject; institutionCode: IECA; basisOfRecord: PreservedSpecimen
- **Type status:** Other material. **Occurrence:** individualCount: 2; lifeStage: adult; **Taxon:** scientificName: Eublemmaaccedensaethiopica (Hacker, 2019); genus: Eublemma; specificEpithet: accedens aethiopica; scientificNameAuthorship: Hacker, 2019; **Location:** country: South Africa; stateProvince: Limpopo; verbatimLocality: Kruger NP, Tsendze river, road S48, 1 km W of Klein Nshawu waterhole; verbatimElevation: 315 m; decimalLatitude: -23.625623; decimalLongitude: 31.470533; **Identification:** identifiedBy: Fernando P. Gaona, Sylvain Delabye; dateIdentified: 2022; **Event:** eventDate: 02/18/2020; habitat: Seasonal river; open savanna grassland with stunted mopane; **Record Level:** type: PhysicalObject; institutionCode: IECA (x1); ZMJU (x1); basisOfRecord: PreservedSpecimen
- **Type status:** Other material. **Occurrence:** individualCount: 3; lifeStage: adult; **Taxon:** scientificName: Eublemmaaccedensaethiopica (Hacker, 2019); genus: Eublemma; specificEpithet: accedens aethiopica; scientificNameAuthorship: Hacker, 2019; **Location:** country: South Africa; stateProvince: Limpopo; verbatimLocality: Kruger NP, Nkulumbeni dam; verbatimElevation: 297 m; decimalLatitude: -23.008383; decimalLongitude: 31.371634; **Identification:** identifiedBy: Fernando P. Gaona, Sylvain Delabye; dateIdentified: 2022; **Event:** eventDate: 02/27/2020; habitat: Open savanna grassland with stunted mopane; **Record Level:** type: PhysicalObject; institutionCode: IECA; basisOfRecord: PreservedSpecimen
- **Type status:** Other material. **Occurrence:** individualCount: 1; lifeStage: adult; **Taxon:** scientificName: Eublemmaaccedensaethiopica (Hacker, 2019); genus: Eublemma; specificEpithet: accedens aethiopica; scientificNameAuthorship: Hacker, 2019; **Location:** country: South Africa; stateProvince: Limpopo; verbatimLocality: Kruger NP, Tinyarhini waterhole; verbatimElevation: 280 m; decimalLatitude: -23.151872; decimalLongitude: 31.475738; **Identification:** identifiedBy: Fernando P. Gaona, Sylvain Delabye; dateIdentified: 2022; **Event:** eventDate: 02/27/2020; habitat: Open savanna grassland with stunted mopane; **Record Level:** type: PhysicalObject; institutionCode: ZMJU; basisOfRecord: PreservedSpecimen
- **Type status:** Other material. **Occurrence:** individualCount: 3; lifeStage: adult; **Taxon:** scientificName: Eublemmaaccedensaethiopica (Hacker, 2019); genus: Eublemma; specificEpithet: accedens aethiopica; scientificNameAuthorship: Hacker, 2019; **Location:** country: South Africa; stateProvince: Limpopo; verbatimLocality: Kruger NP, Nyawutsi waterhole; verbatimElevation: 288 m; decimalLatitude: -23.294739; decimalLongitude: 31.529902; **Identification:** identifiedBy: Fernando P. Gaona, Sylvain Delabye; dateIdentified: 2022; **Event:** eventDate: 02/26/2020; habitat: Seasonal river; Lebombo - low, arid rhyolite hills: Acacia and thorn thickets; **Record Level:** type: PhysicalObject; institutionCode: IECA (x1); ZMJU (x2); basisOfRecord: PreservedSpecimen
- **Type status:** Other material. **Occurrence:** individualCount: 2; lifeStage: adult; **Taxon:** scientificName: Eublemmaaccedensaethiopica (Hacker, 2019); genus: Eublemma; specificEpithet: accedens aethiopica; scientificNameAuthorship: Hacker, 2019; **Location:** country: South Africa; stateProvince: Limpopo; verbatimLocality: Kruger NP, road S50, 2 km MW of Shibavantse lookout; verbatimElevation: 364 m; decimalLatitude: -23.400426; decimalLongitude: 31.531024; **Identification:** identifiedBy: Fernando P. Gaona, Sylvain Delabye; dateIdentified: 2022; **Event:** eventDate: 02/26/2020; habitat: Crest; Lebombo - low, arid rhyolite hills: Acacia and thorn thickets; **Record Level:** type: PhysicalObject; institutionCode: ZMJU; basisOfRecord: PreservedSpecimen
- **Type status:** Other material. **Occurrence:** individualCount: 1; lifeStage: adult; **Taxon:** scientificName: Eublemmaaccedensaethiopica (Hacker, 2019); genus: Eublemma; specificEpithet: accedens aethiopica; scientificNameAuthorship: Hacker, 2019; **Location:** country: South Africa; stateProvince: Limpopo; verbatimLocality: Kruger NP, Shingwedzi river, Historical Site 50; verbatimElevation: 266 m; decimalLatitude: -23.22038; decimalLongitude: 31.552017; **Identification:** identifiedBy: Fernando P. Gaona, Sylvain Delabye; dateIdentified: 2022; **Event:** eventDate: 02/26/2020; habitat: Perennial river; Lebombo - low, arid rhyolite hills: Acacia and thorn thickets; **Record Level:** type: PhysicalObject; institutionCode: ZMJU; basisOfRecord: PreservedSpecimen

#### Description

The detailed diagnosis made by Hacker (2019) enabled identification of this species.

#### Distribution

**Figure 2.**
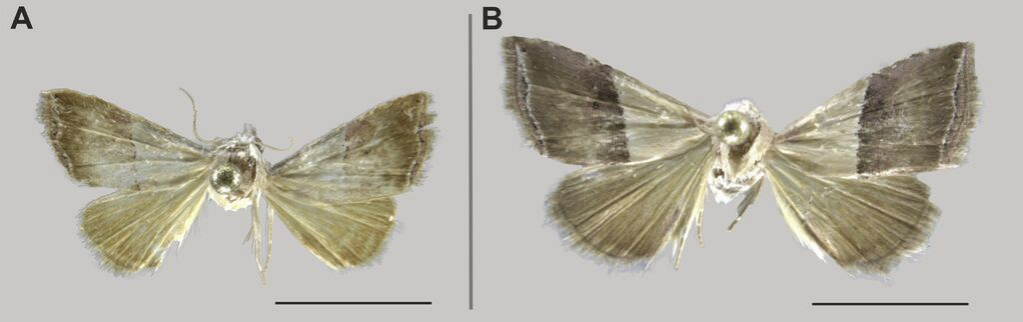
*Eublemmaaccedensaethiopica* Hacker, 2019, South Africa, Kruger National Park; **A** male; **B** female. The scale represents 5 mm.

We report this species for the first time in South Africa. This widespread species’ distribution was previously known to range from West Africa to south-eastern Asia, the Pacific Islands and northern Australia. The African subspecies *E.a.aethiopica* was already known from the Guinean (Nigeria, Guinea, Burkina Faso), Congolian (Democratic Republic of the Congo), Somalian (Ethiopia, Kenya) and Zambezian (Tanzania) biogeographical regions and from Madagascar (De Prins and De Prins 2022). Hacker (2019) suggested that only *Eublemmacaffrorum* (Wallengern, 1860) occurs in southern Africa. However, genitalia examination of our specimens clearly revealed that both species co-occur in KNP. Both species are barely morphologically distinguishable, which could have explained that *E.accedens* was overlooked in southern Africa. Thus, our record extended the southern border of the species’ known distribution range in the continent by over 1,000 km (Fig. 2). Nevertheless, we suppose it could have been overlooked because it is distinguishable only by genitalia dissection (Hacker 2019).

### Eublemma savour

Berio, 1950

7461C531-DF11-5933-9461-8C729368CDD2

Erebidae , Boletobiinae

#### Materials

- **Type status:** Other material. **Occurrence:** individualCount: 1; lifeStage: adult; **Taxon:** scientificName: Eublemmasavour Berio, 1950; **Location:** country: South Africa; stateProvince: Mpumalanga; verbatimLocality: Kruger NP, road H1-6, 1 km SW of Olifantsbad pan; verbatimElevation: 426 m; decimalLatitude: -23.335829; decimalLongitude: 31.325420; **Identification:** identifiedBy: Sylvain Delabye; dateIdentified: 2022; **Event:** eventDate: 24/02/2020; habitat: Crest; mopani dominated woodland savanna on granite: Acacia, Bushwillow and Mopane; **Record Level:** type: PhysicalObject; institutionCode: ZMJU; basisOfRecord: PreservedSpecimen
- **Type status:** Other material. **Occurrence:** individualCount: 1; sex: female; lifeStage: adult; **Taxon:** scientificName: Eublemmasavour Berio, 1950; **Location:** country: South Africa; stateProvince: Limpopo; verbatimLocality: Kruger NP, road H9, close to Misumani river; verbatimElevation: 379 m; decimalLatitude: -23.930505; decimalLongitude: 31.398723; **Identification:** identifiedBy: Sylvain Delabye; dateIdentified: 2022; **Event:** eventDate: 02/12/2019; habitat: Crest; mixed woodland savanna with sweet grazing: Knobthorn and Marula; **Record Level:** type: PhysicalObject; institutionCode: ZMJU; basisOfRecord: PreservedSpecimen
- **Type status:** Other material. **Occurrence:** individualCount: 1; sex: female; lifeStage: adult; **Taxon:** scientificName: Eublemmasavour Berio, 1950; **Location:** country: South Africa; stateProvince: Limpopo; verbatimLocality: Kruger NP, road S47, Nwanedzi river; verbatimElevation: 278 m; decimalLatitude: -23.793044; decimalLongitude: 31.479490; **Identification:** identifiedBy: Sylvain Delabye; dateIdentified: 2022; **Event:** eventDate: 21/02/2020; habitat: Seasonal river; mopani dominated woodland savanna on granite: Acacia, Bushwillow and Mopane; **Record Level:** type: PhysicalObject; institutionCode: ZMJU; basisOfRecord: PreservedSpecimen
- **Type status:** Other material. **Occurrence:** individualCount: 1; sex: female; lifeStage: adult; **Taxon:** scientificName: Eublemmasavour Berio, 1950; **Location:** country: South Africa; stateProvince: Limpopo; verbatimLocality: Kruger NP, Letaba river, 6 km N of Letaba camp; verbatimElevation: 245 m; decimalLatitude: -23.811079; decimalLongitude: 31.580949; **Identification:** identifiedBy: Sylvain Delabye; dateIdentified: 2022; **Event:** eventDate: 18/02/2020; habitat: Perennial river; open savanna grassland with stunted mopane; **Record Level:** type: PhysicalObject; institutionCode: ZMJU; basisOfRecord: PreservedSpecimen
- **Type status:** Other material. **Occurrence:** individualCount: 1; sex: female; lifeStage: adult; **Taxon:** scientificName: Eublemmasavour Berio, 1950; **Location:** country: South Africa; stateProvince: Limpopo; verbatimLocality: Kruger NP, road H1-5, 11 km S of Letaba camp; verbatimElevation: 301 m; decimalLatitude: -23.930371; decimalLongitude: 31.600825; **Identification:** identifiedBy: Sylvain Delabye; dateIdentified: 2022; **Event:** eventDate: 20/02/2020; habitat: Crest; open savanna grassland with stunted mopane; **Record Level:** type: PhysicalObject; institutionCode: ZMJU; basisOfRecord: PreservedSpecimen
- **Type status:** Other material. **Occurrence:** individualCount: 1; sex: male; lifeStage: adult; **Taxon:** scientificName: Eublemmasavour Berio, 1950; **Location:** country: South Africa; stateProvince: Limpopo; verbatimLocality: Kruger NP, Letaba river, 10 km E of Letaba camp; verbatimElevation: 217 m; decimalLatitude: -23.844905; decimalLongitude: 31.641514; **Identification:** identifiedBy: Sylvain Delabye; dateIdentified: 2022; **Event:** eventDate: 20/02/2020; habitat: Perennial river; open savanna grassland with stunted mopane; **Record Level:** type: PhysicalObject; institutionCode: ZMJU; basisOfRecord: PreservedSpecimen
- **Type status:** Other material. **Occurrence:** individualCount: 2; lifeStage: adult; **Taxon:** scientificName: Eublemmasavour Berio, 1950; **Location:** country: South Africa; stateProvince: Mpumalanga; verbatimLocality: Kruger NP, Crocodile river, Crocodile Bridge; verbatimElevation: 183 m; decimalLatitude: -25.363011; decimalLongitude: 31.891832; **Identification:** identifiedBy: Sylvain Delabye; dateIdentified: 2022; **Event:** eventDate: 07/03/2019; habitat: Perennial river; open savanna grassland on basalt; **Record Level:** type: PhysicalObject; institutionCode: ZMJU; basisOfRecord: PreservedSpecimen
- **Type status:** Other material. **Occurrence:** individualCount: 1; lifeStage: adult; **Taxon:** scientificName: Eublemmasavour Berio, 1950; **Location:** country: South Africa; stateProvince: Mpumalanga; verbatimLocality: Kruger NP, Mnondozi river, pan 1 km S of Hlwehlwe; verbatimElevation: 188 m; decimalLatitude: -25.067375; decimalLongitude: 31.973211; **Identification:** identifiedBy: Sylvain Delabye; dateIdentified: 2022; **Event:** eventDate: 05/03/2019; habitat: Seasonal river; open savanna grassland on basalt; **Record Level:** type: PhysicalObject; institutionCode: ZMJU; basisOfRecord: PreservedSpecimen
- **Type status:** Other material. **Occurrence:** individualCount: 1; lifeStage: adult; **Taxon:** scientificName: Eublemmasavour Berio, 1950; **Location:** country: South Africa; stateProvince: Mpumalanga; verbatimLocality: Kruger NP, Vurhami river, road H4-2; verbatimElevation: 219 m; decimalLatitude: -25.246385; decimalLongitude: 31.847472; **Identification:** identifiedBy: Sylvain Delabye; dateIdentified: 2022; **Event:** eventDate: 07/03/2019; habitat: Season river; wooded savanna on shale: Bushwillow, Leadwood, Marula and Knobthorn trees, and thorn thickets; **Record Level:** type: PhysicalObject; institutionCode: ZMJU; basisOfRecord: PreservedSpecimen

#### Description

The identification was based on Hacker (2019).

#### Distribution

**Figure 3.**
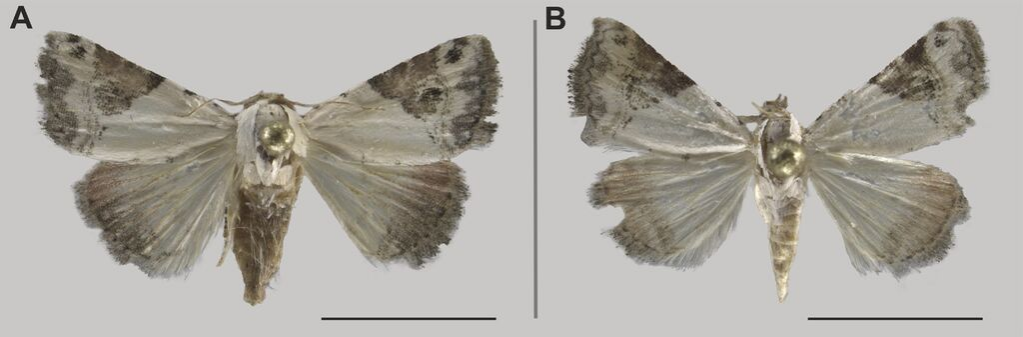
*Eublemmasavour* Berio, 1950, South Africa, Kruger National Park; **A** male; **B** female. The scale represents 5 mm.

We report this species for the first time in South Africa. It was already known from several countries in the Guinean (Burkina Faso, Nigeria), Somalian (Sudan, Ethiopia, Eritrea and Kenya), Zambezian and Southern African biogeographical regions, including Zimbabwe bordering with South Africa (Hacker 2019). Its occurrence in KNP is, therefore, not surprising (Fig. 3).

### Beriodesma determinata

(Wallengren, 1863)

09F1E226-D436-587D-A4D2-5D0DF7D338BD

Erebidae , Erebinae

#### Materials

- **Type status:** Other material. **Occurrence:** individualCount: 2; sex: 1 male, 1 female; lifeStage: adult; **Taxon:** scientificName: Beriodesmadeterminata (Wallengren, 1863); **Location:** country: South Africa; stateProvince: Limpopo; verbatimLocality: Kruger NP, Tsendze river, road S48, 1 km W of Klein Nshawu waterhole; verbatimElevation: 315 m; decimalLatitude: -23.625623; decimalLongitude: 31.470533; **Identification:** identifiedBy: Sylvain Delabye; dateIdentified: 2022; **Event:** eventDate: 18/02/2020; habitat: Seasonal river; open savanna grassland with stunted mopane; **Record Level:** type: PhysicalObject; institutionCode: ZMJU; basisOfRecord: PreservedSpecimen
- **Type status:** Other material. **Occurrence:** individualCount: 1; sex: female; lifeStage: adult; **Taxon:** scientificName: Beriodesmadeterminata (Wallengren, 1863); **Location:** country: South Africa; stateProvince: Limpopo; verbatimLocality: Kruger NP, road H1-5, 11 km S of Letaba camp; verbatimElevation: 301 m; decimalLatitude: -23.930371; decimalLongitude: 31.600825; **Identification:** identifiedBy: Sylvain Delabye; dateIdentified: 2022; **Event:** eventDate: 20/02/2020; habitat: Crest; open savanna grassland with stunted mopane; **Record Level:** type: PhysicalObject; institutionCode: ZMJU; basisOfRecord: PreservedSpecimen
- **Type status:** Other material. **Occurrence:** individualCount: 1; sex: female; lifeStage: adult; **Taxon:** scientificName: Beriodesmadeterminata (Wallengren, 1863); **Location:** country: South Africa; stateProvince: Limpopo; verbatimLocality: Kruger NP, road S50, 2 km MW of Shibavantse lookout; verbatimElevation: 364 m; decimalLatitude: -23.400426; decimalLongitude: 31.531024; **Identification:** identifiedBy: Sylvain Delabye; dateIdentified: 2022; **Event:** eventDate: 26/02/2020; habitat: Crest; Lebombo - low, arid rhyolite hills: Acacia and thorn thickets; **Record Level:** type: PhysicalObject; institutionCode: ZMJU; basisOfRecord: PreservedSpecimen
- **Type status:** Other material. **Occurrence:** individualCount: 1; sex: female; lifeStage: adult; **Taxon:** scientificName: Beriodesmadeterminata (Wallengren, 1863); **Location:** country: South Africa; stateProvince: Limpopo; verbatimLocality: Kruger NP, Letaba river, 5 km E of Road H14; verbatimElevation: 278 m; decimalLatitude: -23.757038; decimalLongitude: 31.392944; **Identification:** identifiedBy: Sylvain Delabye; dateIdentified: 2022; **Event:** eventDate: 19/02/2020; habitat: Perennial river; mopani dominated woodland on granite: Acacia, Bushwillow and Mopane; **Record Level:** type: PhysicalObject; institutionCode: ZMJU; basisOfRecord: PreservedSpecimen
- **Type status:** Other material. **Occurrence:** individualCount: 1; sex: female; lifeStage: adult; **Taxon:** scientificName: Beriodesmadeterminata (Wallengren, 1863); **Location:** country: South Africa; stateProvince: Mpumalanga; verbatimLocality: Kruger NP, N’wanetzi river, road S100, 2 km W of Nsasane waterhole; verbatimElevation: 228 m; decimalLatitude: -24.381219; decimalLongitude: 31.886093; **Identification:** identifiedBy: Sylvain Delabye; dateIdentified: 2022; **Event:** eventDate: 02/03/2019; habitat: Seasonal river; Olifants rugged veld: mixed thornveld and woodland savanna; **Record Level:** type: PhysicalObject; institutionCode: ZMJU; basisOfRecord: PreservedSpecimen
- **Type status:** Other material. **Occurrence:** individualCount: 1; lifeStage: adult; **Taxon:** scientificName: Beriodesmadeterminata (Wallengren, 1863); **Location:** country: South Africa; stateProvince: Mpumalanga; verbatimLocality: Kruger NP, Silolweni river, road H1-2, 1 km S of Silolweni dam; verbatimElevation: 284 m; decimalLatitude: -24.820865; decimalLongitude: 31.844014; **Identification:** identifiedBy: Sylvain Delabye; dateIdentified: 2022; **Event:** eventDate: 26/02/2019; habitat: Seasonal river; open savanna grassland on basalt; **Record Level:** type: PhysicalObject; institutionCode: ZMJU; basisOfRecord: PreservedSpecimen
- **Type status:** Other material. **Occurrence:** individualCount: 1; lifeStage: adult; **Taxon:** scientificName: Beriodesmadeterminata (Wallengren, 1863); **Location:** country: South Africa; stateProvince: Mpumalanga; verbatimLocality: Kruger NP, Vurhami river, road H4-2; verbatimElevation: 219 m; decimalLatitude: -25.246385; decimalLongitude: 31.847472; **Identification:** identifiedBy: Sylvain Delabye; dateIdentified: 2022; **Event:** eventDate: 07/03/2019; habitat: Season river; wooded savanna on shale: Bushwillow, Leadwood, Marula and Knobthorn trees, and thorn thickets; **Record Level:** type: PhysicalObject; institutionCode: ZMJU; basisOfRecord: PreservedSpecimen
- **Type status:** Other material. **Occurrence:** individualCount: 1; sex: female; lifeStage: adult; **Taxon:** scientificName: Beriodesmadeterminata (Wallengren, 1863); **Location:** country: South Africa; stateProvince: Mpumalanga; verbatimLocality: Kruger NP, N’waswitsontso river, road S125, 1 km NW of Mhisanamond waterhole; verbatimElevation: 329 m; decimalLatitude: -24.600415; decimalLongitude: 31.687177; **Identification:** identifiedBy: Sylvain Delabye; dateIdentified: 2022; **Event:** eventDate: 03/03/2019; habitat: Perennial river; mixed thorn and Marula woodland on granite: Bushwillow, Acacia and Marula; **Record Level:** type: PhysicalObject; institutionCode: ZMJU; basisOfRecord: PreservedSpecimen
- **Type status:** Other material. **Occurrence:** individualCount: 1; lifeStage: adult; **Taxon:** scientificName: Beriodesmadeterminata (Wallengren, 1863); **Location:** country: South Africa; stateProvince: Mpumalanga; verbatimLocality: Kruger NP, Sabie river, 2 km S of N'wanghandze waterhole; verbatimElevation: 152 m; decimalLatitude: -25.158984; decimalLongitude: 31.998265; **Identification:** identifiedBy: Sylvain Delabye; dateIdentified: 2022; **Event:** eventDate: 05/03/2019; habitat: Perennial river; Lebombo - low, arid rhyolite hills: Acacia and thorn thickets; **Record Level:** type: PhysicalObject; institutionCode: ZMJU; basisOfRecord: PreservedSpecimen

#### Description

The identification was mainly based on Hacker (2016) and on photographs in De Prins and De Prins (2022).

#### Distribution

**Figure 4.**
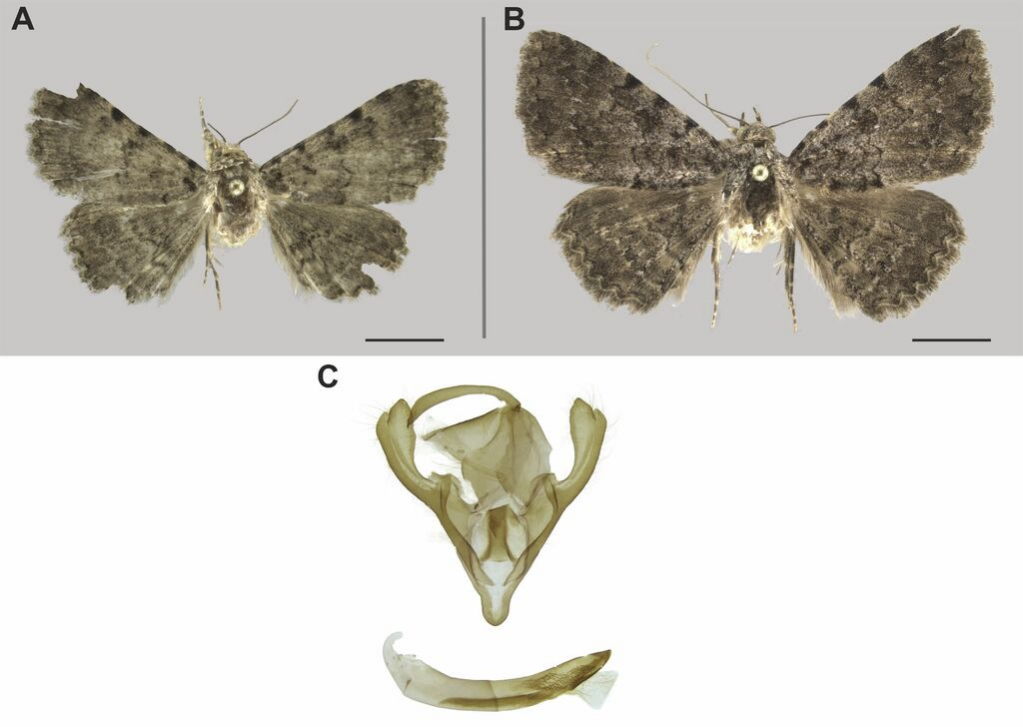
*Beriodesmadeterminata* (Wallengren, 1863), South Africa, Kruger National Park; **A** male; **B** female; **C** male genitalia.The scale represents 5 mm.

We report this species for the first time in South Africa. The known distribution of this species already included the Zambezian (Tanzania and Zimbabwe), Somalian (Kenya) and Southern African (Namibia) biogeographic regions (Hacker 2016, De Prins and De Prins 2022). The extension of its known distribution to KNP is not surprising (Fig. 4).

### Heteropalpia exarata

(Mabille, 1890)

A07C7A35-1ACA-50DB-ADE6-356CC8898882

Erebidae , Erebinae

#### Materials

- **Type status:** Other material. **Occurrence:** individualCount: 1; sex: male; lifeStage: adult; **Taxon:** scientificName: Heteropalpiaexarata (Mabille, 1890); **Location:** country: South Africa; stateProvince: Mpumalanga; verbatimLocality: Kruger NP, 1 km NW of Balule camp; verbatimElevation: 269 m; decimalLatitude: -24.053562; decimalLongitude: 31.722598; **Identification:** identifiedBy: Sylvain Delabye; dateIdentified: 2022; **Event:** eventDate: 20/11/2019; habitat: Perennial river; open savanna grassland with stunted mopane; **Record Level:** type: PhysicalObject; institutionCode: ZMJU; basisOfRecord: PreservedSpecimen
- **Type status:** Other material. **Occurrence:** individualCount: 1; sex: male; lifeStage: adult; **Taxon:** scientificName: Heteropalpiaexarata (Mabille, 1890); **Location:** country: South Africa; stateProvince: Limpopo; verbatimLocality: Kruger NP, Road S56, Mphongolo river, 1 km N of Ribye waterhole; verbatimElevation: 314 m; decimalLatitude: -22.991653; decimalLongitude: 31.270644; **Identification:** identifiedBy: Sylvain Delabye; dateIdentified: 2022; **Event:** eventDate: 25/02/2020; habitat: Perennial river; open savanna grassland with stunted mopane; **Record Level:** type: PhysicalObject; institutionCode: ZMJU; basisOfRecord: PreservedSpecimen
- **Type status:** Other material. **Occurrence:** individualCount: 1; sex: female; lifeStage: adult; **Taxon:** scientificName: Heteropalpiaexarata (Mabille, 1890); **Location:** country: South Africa; stateProvince: Mpumalanga; verbatimLocality: Kruger NP, Road H1-3, 500m W of Marheya waterhole; verbatimElevation: 333 m; decimalLatitude: -24.565596; decimalLongitude: 31.774760; **Identification:** identifiedBy: Sylvain Delabye; dateIdentified: 2022; **Event:** eventDate: 01/03/2019; habitat: Crest; wooded savanna on shale: Bushwillow, Leadwood, Marula, Knobthorn trees, and thorn thickets; **Record Level:** type: PhysicalObject; institutionCode: ZMJU; basisOfRecord: PreservedSpecimen
- **Type status:** Other material. **Occurrence:** individualCount: 1; sex: male; lifeStage: adult; **Taxon:** scientificName: Heteropalpiaexarata (Mabille, 1890); **Location:** country: South Africa; stateProvince: Mpumalanga; verbatimLocality: Kruger NP, Crocodile river, road S114, 5 km N of Malelane gate; verbatimElevation: 300 m; decimalLatitude: -25.437282; decimalLongitude: 31.528655; **Identification:** identifiedBy: Sylvain Delabye; dateIdentified: 2022; **Event:** eventDate: 25/02/2019; habitat: Perennial river; mixed woodland savanna and thorn thickets: Tamboti, Acacia, Marula and Bushwillow; **Record Level:** type: PhysicalObject; institutionCode: ZMJU; basisOfRecord: PreservedSpecimen
- **Type status:** Other material. **Occurrence:** individualCount: 1; sex: female; lifeStage: adult; **Taxon:** scientificName: Heteropalpiaexarata (Mabille, 1890); **Location:** country: South Africa; stateProvince: Mpumalanga; verbatimLocality: Kruger NP, road S114, 3 km N of Historical Site 20; verbatimElevation: 366 m; decimalLatitude: -25.070953; decimalLongitude: 31.599270; **Identification:** identifiedBy: Sylvain Delabye; dateIdentified: 2022; **Event:** eventDate: 27/02/2019; habitat: Crest; mixed thorn and Marula woodland savanna on granite: Bushwillow, Acacia and Marula; **Record Level:** type: PhysicalObject; institutionCode: ZMJU; basisOfRecord: PreservedSpecimen
- **Type status:** Other material. **Occurrence:** individualCount: 1; lifeStage: adult; **Taxon:** scientificName: Heteropalpiaexarata (Mabille, 1890); **Location:** country: South Africa; stateProvince: Mpumalanga; verbatimLocality: Kruger NP, Sabie river, Lookout on S79 road; verbatimElevation: 200 m; decimalLatitude: -25.064099; decimalLongitude: 31.825735; **Identification:** identifiedBy: Sylvain Delabye; dateIdentified: 2022; **Event:** eventDate: 24/02/2019; habitat: Perennial river; mixed woodland savanna and thorn thickets: Tamboti, Acacia, Marula and Bushwillow; **Record Level:** type: PhysicalObject; institutionCode: ZMJU; basisOfRecord: PreservedSpecimen

#### Description

The identification was mainly based on Hacker (2016) and on photographs in De Prins and De Prins (2022).

#### Distribution

**Figure 5.**
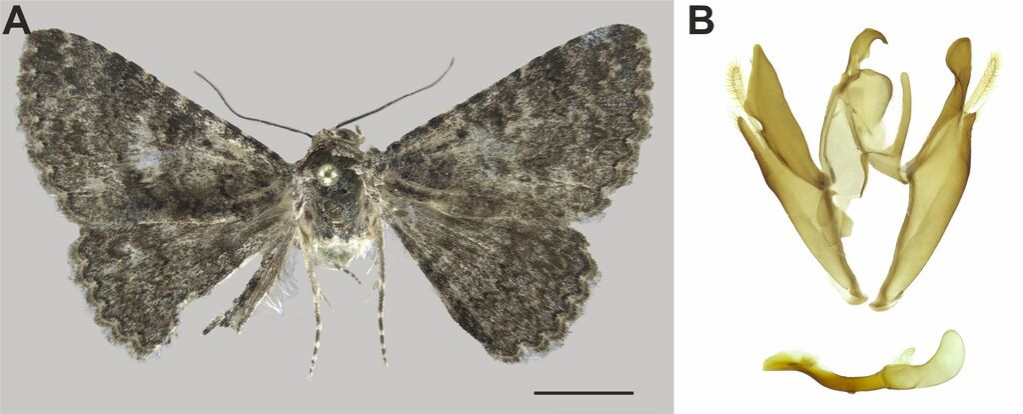
*Heteropalpiaexarata* (Mabille, 1890), South Africa, Kruger National Park; **A** male; **B** male genitalia. The scale represents 5 mm.

We report this species for the first time in South Africa. This widespread species was already reported from the Sahel-Saharan (Mauritania), Guinean (Burkina Faso, Nigeria), Sudanian (Chad, Sudan), Somalian (Kenya, Ethiopa, Eritrea, Somalia), Zambezian and Southern African regions, including Zimbabwe bordering with South Africa (Hacker 2016, De Prins and De Prins 2022) (Fig. 5).

### Ozarba atrisigna

(Hampson, 1910)

7DEC216C-832E-572D-A62B-AB9283BB7E80

Noctuidae , Eustrotiinae

#### Materials

- **Type status:** Other material. **Occurrence:** individualCount: 1; lifeStage: adult; **Taxon:** scientificName: Ozarbaatrisigna (Hampson, 1910); **Location:** country: South Africa; stateProvince: Mpumalanga; verbatimLocality: Kruger NP, Sabie river, 2 km S of N'wanghandze waterhole; verbatimElevation: 152 m; decimalLatitude: -25.158984; decimalLongitude: 31.998265; **Identification:** identifiedBy: Sylvain Delabye; dateIdentified: 2022; **Event:** eventDate: 05/03/2019; habitat: Perennial river; Lebombo - low, arid rhyolite hills: Acacia and thorn thickets; **Record Level:** type: PhysicalObject; institutionCode: IECA; basisOfRecord: PreservedSpecimen
- **Type status:** Other material. **Occurrence:** individualCount: 1; lifeStage: adult; **Taxon:** scientificName: Ozarbaatrisigna (Hampson, 1910); **Location:** country: South Africa; stateProvince: Limpopo; verbatimLocality: Kruger NP, Letaba river, 5 km E of Road H14; verbatimElevation: 278 m; decimalLatitude: -23.757038; decimalLongitude: 31.392944; **Identification:** identifiedBy: Sylvain Delabye; dateIdentified: 2022; **Event:** eventDate: 19/02/2020; habitat: Perennial river; mopani dominated woodland savanna on granite: Acacia, Bushwillow and Mopane; **Record Level:** type: PhysicalObject; institutionCode: IECA; basisOfRecord: PreservedSpecimen
- **Type status:** Other material. **Occurrence:** individualCount: 1; lifeStage: adult; **Taxon:** scientificName: Ozarbaatrisigna (Hampson, 1910); **Location:** country: South Africa; stateProvince: Limpopo; verbatimLocality: Kruger NP, Road S133, 6 km S of Jumbo waterhole; verbatimElevation: 365 m; decimalLatitude: -23.844016; decimalLongitude: 31.365157; **Identification:** identifiedBy: Sylvain Delabye; dateIdentified: 2022; **Event:** eventDate: 19/02/2020; habitat: Crest; mixed woodland savanna with sweet grazing: Knobthorn and Marula; **Record Level:** type: PhysicalObject; institutionCode: IECA; basisOfRecord: PreservedSpecimen
- **Type status:** Other material. **Occurrence:** individualCount: 5; sex: 2 males, 3 females; lifeStage: adult; **Taxon:** scientificName: Ozarbaatrisigna (Hampson, 1910); **Location:** country: South Africa; stateProvince: Mpumalanga; verbatimLocality: Kruger NP, Mnondozi river, pan 1 km S of Hlwehlwe; verbatimElevation: 188 m; decimalLatitude: -25.067375; decimalLongitude: 31.973211; **Identification:** identifiedBy: Sylvain Delabye; dateIdentified: 2022; **Event:** eventDate: 05/03/2019; habitat: Seasonal river; open savanna grassland on basalt; **Record Level:** type: PhysicalObject; institutionCode: ZMJU; basisOfRecord: PreservedSpecimen
- **Type status:** Other material. **Occurrence:** individualCount: 1; sex: female; lifeStage: adult; **Taxon:** scientificName: Ozarbaatrisigna (Hampson, 1910); **Location:** country: South Africa; stateProvince: Mpumalanga; verbatimLocality: Kruger NP, Vurhami river, road H4-2; verbatimElevation: 219 m; decimalLatitude: -25.246385; decimalLongitude: 31.847472; **Identification:** identifiedBy: Sylvain Delabye; dateIdentified: 2022; **Event:** eventDate: 07/03/2019; habitat: Season river; wooded savanna on shale: Bushwillow, Leadwood, Marula and Knobthorn trees, and thorn thickets; **Record Level:** type: PhysicalObject; institutionCode: IECA; basisOfRecord: PreservedSpecimen
- **Type status:** Other material. **Occurrence:** individualCount: 1; lifeStage: adult; **Taxon:** scientificName: Ozarbaatrisigna (Hampson, 1910); **Location:** country: South Africa; stateProvince: Limpopo; verbatimLocality: Kruger NP, Road S47, Nwanedzi river; verbatimElevation: 278 m; decimalLatitude: -23.793044; decimalLongitude: 31.479490; **Identification:** identifiedBy: Sylvain Delabye; dateIdentified: 2022; **Event:** eventDate: 21/02/2020; habitat: Seasonal river; mopani dominated woodland savanna on granite: Acacia, Bushwillow and Mopane; **Record Level:** type: PhysicalObject; institutionCode: IECA; basisOfRecord: PreservedSpecimen
- **Type status:** Other material. **Occurrence:** individualCount: 2; lifeStage: adult; **Taxon:** scientificName: Ozarbaatrisigna (Hampson, 1910); **Location:** country: South Africa; stateProvince: Mpumalanga; verbatimLocality: Kruger NP, Sweni Bird Hide; verbatimElevation: 187 m; decimalLatitude: -24.474196; decimalLongitude: 31.972276; **Identification:** identifiedBy: Sylvain Delabye; dateIdentified: 2022; **Event:** eventDate: 02/03/2019; habitat: Perennial river; rugged veld: mixed thronveld and woodland savanna; **Record Level:** type: PhysicalObject; institutionCode: IECA; basisOfRecord: PreservedSpecimen
- **Type status:** Other material. **Occurrence:** individualCount: 2; lifeStage: adult; **Taxon:** scientificName: Ozarbaatrisigna (Hampson, 1910); **Location:** country: South Africa; stateProvince: Mpumalanga; verbatimLocality: Kruger NP, Gayisenga waterhole; verbatimElevation: 256 m; decimalLatitude: -25.270645; decimalLongitude: 31.756561; **Identification:** identifiedBy: Sylvain Delabye; dateIdentified: 2022; **Event:** eventDate: 06/03/2019; habitat: Seasonal river; mixed thorn and Marula woodland savanna on granite: Bushwillow, Acacia and Marula; **Record Level:** type: PhysicalObject; institutionCode: IECA; basisOfRecord: PreservedSpecimen
- **Type status:** Other material. **Occurrence:** individualCount: 2; lifeStage: adult; **Taxon:** scientificName: Ozarbaatrisigna (Hampson, 1910); **Location:** country: South Africa; stateProvince: Limpopo; verbatimLocality: Kruger NP, Road S47, Nwanedzi river; verbatimElevation: 278 m; decimalLatitude: -23.793044; decimalLongitude: 31.479490; **Identification:** identifiedBy: Sylvain Delabye; dateIdentified: 2022; **Event:** eventDate: 02/12/2019; habitat: Seasonal river; mopani dominated woodlandsavanna on granite: Acacia, Bushwillow and Mopane; **Record Level:** type: PhysicalObject; institutionCode: IECA; basisOfRecord: PreservedSpecimen
- **Type status:** Other material. **Occurrence:** individualCount: 1; lifeStage: adult; **Taxon:** scientificName: Ozarbaatrisigna (Hampson, 1910); **Location:** country: South Africa; stateProvince: Mpumalanga; verbatimLocality: Kruger NP, Sabie river, road H4-1, 7.5 km E fo Skukuza camp; verbatimElevation: 265 m; decimalLatitude: -24.973743; decimalLongitude: 31.661434; **Identification:** identifiedBy: Sylvain Delabye; dateIdentified: 2022; **Event:** eventDate: 27/02/2019; habitat: Perennial river; mixed woodland savanna and thorn thickets: Tamboti, Acacia, Marula and Bushwillow; **Record Level:** type: PhysicalObject; institutionCode: IECA; basisOfRecord: PreservedSpecimen
- **Type status:** Other material. **Occurrence:** individualCount: 1; lifeStage: adult; **Taxon:** scientificName: Ozarbaatrisigna (Hampson, 1910); **Location:** country: South Africa; stateProvince: Mpumalanga; verbatimLocality: Kruger NP, Sabie river, 2 km S of N'wanghandze waterhole; verbatimElevation: 152 m; decimalLatitude: -25.158984; decimalLongitude: 31.998265; **Identification:** identifiedBy: Sylvain Delabye; dateIdentified: 2022; **Event:** eventDate: 09/11/2018; habitat: Perennial river; Lebombo - low, arid rhyolite hills: Acacia and thorn thickets; **Record Level:** type: PhysicalObject; institutionCode: IECA; basisOfRecord: PreservedSpecimen
- **Type status:** Other material. **Occurrence:** individualCount: 1; sex: male; lifeStage: adult; **Taxon:** scientificName: Ozarbaatrisigna (Hampson, 1910); **Location:** country: South Africa; stateProvince: Mpumalanga; verbatimLocality: Kruger NP, Road S122, 4 km E of Hillside waterhole; verbatimElevation: 281 m; decimalLatitude: -24.973976; decimalLongitude: 31.928260; **Identification:** identifiedBy: Sylvain Delabye; dateIdentified: 2022; **Event:** eventDate: 05/03/2019; habitat: Crest; open savanna grassland on basalt; **Record Level:** type: PhysicalObject; institutionCode: IECA; basisOfRecord: PreservedSpecimen
- **Type status:** Other material. **Occurrence:** individualCount: 1; lifeStage: adult; **Taxon:** scientificName: Ozarbaatrisigna (Hampson, 1910); **Location:** country: South Africa; stateProvince: Mpumalanga; verbatimLocality: Kruger NP, road S36, 5 km NE of Hlanguleni picnic site; verbatimElevation: 333 m; decimalLatitude: -24.681967; decimalLongitude: 31.666664; **Identification:** identifiedBy: Sylvain Delabye; dateIdentified: 2022; **Event:** eventDate: 03/03/2019; habitat: Crest; mixed thorn and Marula woodland savanna on granite: Bushwillow, Acacia and Marula; **Record Level:** type: PhysicalObject; institutionCode: IECA; basisOfRecord: PreservedSpecimen
- **Type status:** Other material. **Occurrence:** individualCount: 1; lifeStage: adult; **Taxon:** scientificName: Ozarbaatrisigna (Hampson, 1910); **Location:** country: South Africa; stateProvince: Limpopo; verbatimLocality: Kruger NP, Shingwedzi river, Historical Site 50; verbatimElevation: 266 m; decimalLatitude: -23.220380; decimalLongitude: 31.552017; **Identification:** identifiedBy: Sylvain Delabye; dateIdentified: 2022; **Event:** eventDate: 26/02/2020; habitat: Perennial river; Lebombo - low, arid rhyolite hills: Acacia and thorn thickets; **Record Level:** type: PhysicalObject; institutionCode: IECA; basisOfRecord: PreservedSpecimen
- **Type status:** Other material. **Occurrence:** individualCount: 1; sex: female; lifeStage: adult; **Taxon:** scientificName: Ozarbaatrisigna (Hampson, 1910); **Location:** country: South Africa; stateProvince: Mpumalanga; verbatimLocality: Kruger NP, N’waswitsontso river, road S125, 1 km NW of Mhisanamond waterhole; verbatimElevation: 329 m; decimalLatitude: -24.600415; decimalLongitude: 31.687177; **Identification:** identifiedBy: Sylvain Delabye; dateIdentified: 2022; **Event:** eventDate: 03/03/2019; habitat: Perennial river; mixed thorn and Marula woodland savanna on granite: Bushwillow, Acacia and Marula; **Record Level:** type: PhysicalObject; institutionCode: IECA; basisOfRecord: PreservedSpecimen
- **Type status:** Other material. **Occurrence:** individualCount: 1; lifeStage: adult; **Taxon:** scientificName: Ozarbaatrisigna (Hampson, 1910); **Location:** country: South Africa; stateProvince: Limpopo; verbatimLocality: Kruger NP, Stapelkop dam; verbatimElevation: 362 m; decimalLatitude: -23.594645; decimalLongitude: 31.254819; **Identification:** identifiedBy: Sylvain Delabye; dateIdentified: 2022; **Event:** eventDate: 30/11/2019; habitat: Perennial river; mopani dominated woodland savanna on granite: Acacia, Bushwillow and Mopane; **Record Level:** type: PhysicalObject; institutionCode: IECA; basisOfRecord: PreservedSpecimen
- **Type status:** Other material. **Occurrence:** individualCount: 1; sex: female; lifeStage: adult; **Taxon:** scientificName: Ozarbaatrisigna (Hampson, 1910); **Location:** country: South Africa; stateProvince: Limpopo; verbatimLocality: Kruger NP, Nhlanganini dam; verbatimElevation: 327 m; decimalLatitude: -23.933918; decimalLongitude: 31.494673; **Identification:** identifiedBy: Sylvain Delabye; dateIdentified: 2022; **Event:** eventDate: 21/02/2020; habitat: Perennial river; mixed woodland savanna with sweet grazing: Knobthorn and Marula; **Record Level:** type: PhysicalObject; institutionCode: IECA; basisOfRecord: PreservedSpecimen
- **Type status:** Other material. **Occurrence:** individualCount: 1; lifeStage: adult; **Taxon:** scientificName: Ozarbaatrisigna (Hampson, 1910); **Location:** country: South Africa; stateProvince: Mpumalanga; verbatimLocality: Kruger NP, N’wanetzi river, road S100, 2 km W of Nsasane waterhole; verbatimElevation: 228 m; decimalLatitude: -24.381219; decimalLongitude: 31.886093; **Identification:** identifiedBy: Sylvain Delabye; dateIdentified: 2022; **Event:** eventDate: 02/03/2019; habitat: Seasonal river; rugged veld: mixed thornveld and woodland savanna; **Record Level:** type: PhysicalObject; institutionCode: IECA; basisOfRecord: PreservedSpecimen
- **Type status:** Other material. **Occurrence:** individualCount: 1; lifeStage: adult; **Taxon:** scientificName: Ozarbaatrisigna (Hampson, 1910); **Location:** country: South Africa; stateProvince: Limpopo; verbatimLocality: Kruger NP, road H1-5, 11 km S of Letaba camp; verbatimElevation: 301 m; decimalLatitude: -23.930371; decimalLongitude: 31.600825; **Identification:** identifiedBy: Sylvain Delabye; dateIdentified: 2022; **Event:** eventDate: 20/02/2020; habitat: Crest; open savanna grassland with stunted mopane; **Record Level:** type: PhysicalObject; institutionCode: IECA; basisOfRecord: PreservedSpecimen

#### Description

The identification was based on Hacker (2016).

#### Distribution

**Figure 6.**
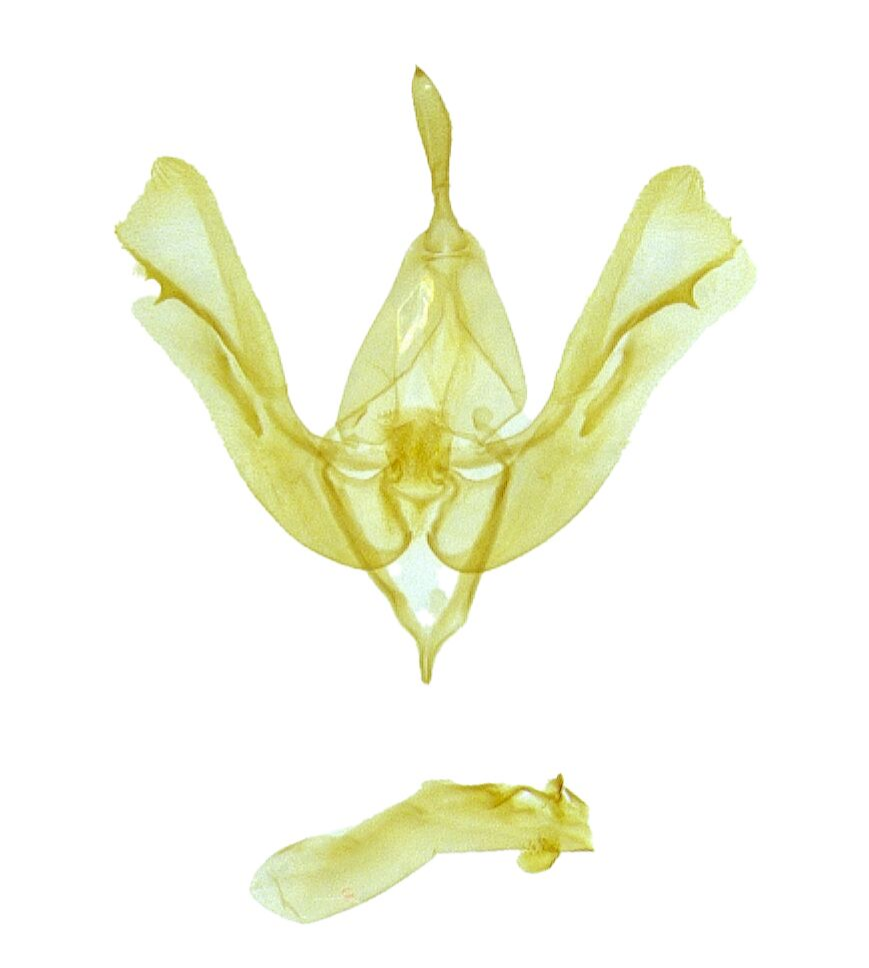
*Ozarbaatrisigna* (Hampson, 1910), male genitalia, South Africa, Kruger National Park.

We report this species for the first time in South Africa. The species was already known from the Zambezian (Tanzania) and Southern African (Namibia) biogeographic regions (Mey 2011, Hacker 2016). Nevertheless, our records substantially extended its known distribution (Fig. 6).

### Ozarba berioi

Hacker, 2016

D169FABB-960E-55F8-ABF4-17B2891F7235

Noctuidae , Eustrotiinae

#### Materials

- **Type status:** Other material. **Occurrence:** individualCount: 1; sex: male; lifeStage: adult; **Taxon:** scientificName: Ozarbaberioi Hacker, 2016; **Location:** country: South Africa; stateProvince: Limpopo; verbatimLocality: Kruger NP, management track at Babalala; verbatimElevation: 357 m; decimalLatitude: -22.898804; decimalLongitude: 31.284004; **Identification:** identifiedBy: Sylvain Delabye; dateIdentified: 2022; **Event:** eventDate: 25/02/2020; habitat: Crest; open savanna grassland with stunted mopane; **Record Level:** type: PhysicalObject; institutionCode: ZMJU; basisOfRecord: PreservedSpecimen
- **Type status:** Other material. **Occurrence:** individualCount: 1; sex: male; lifeStage: adult; **Taxon:** scientificName: Ozarbaberioi Hacker, 2016; **Location:** country: South Africa; stateProvince: Limpopo; verbatimLocality: Kruger NP, Nyawutsi waterhole; verbatimElevation: 288 m; decimalLatitude: -23.294739; decimalLongitude: 31.529902; **Identification:** identifiedBy: Sylvain Delabye; dateIdentified: 2022; **Event:** eventDate: 26/02/2020; habitat: Seasonal river; Lebombo - low, arid rhyolite hills: Acacia and thorn thickets; **Record Level:** type: PhysicalObject; institutionCode: ZMJU; basisOfRecord: PreservedSpecimen
- **Type status:** Other material. **Occurrence:** individualCount: 1; sex: female; lifeStage: adult; **Taxon:** scientificName: Ozarbaberioi Hacker, 2016; **Location:** country: South Africa; stateProvince: Limpopo; verbatimLocality: Kruger NP, road H1-5, 11 km S of Letaba camp; verbatimElevation: 301 m; decimalLatitude: -23.930371; decimalLongitude: 31.600825; **Identification:** identifiedBy: Sylvain Delabye; dateIdentified: 2022; **Event:** eventDate: 20/02/2020; habitat: Crest; open savanna grassland with stunted mopane; **Record Level:** type: PhysicalObject; institutionCode: ZMJU; basisOfRecord: PreservedSpecimen
- **Type status:** Other material. **Occurrence:** individualCount: 1; sex: female; lifeStage: adult; **Taxon:** scientificName: Ozarbaberioi Hacker, 2016; **Location:** country: South Africa; stateProvince: Limpopo; verbatimLocality: Kruger NP, Nkokhodzi river, road H1-6; verbatimElevation: 358 m; decimalLatitude: -23.239622; decimalLongitude: 31.312995; **Identification:** identifiedBy: Sylvain Delabye; dateIdentified: 2022; **Event:** eventDate: 26/11/2019; habitat: Seasonal river; mopani dominated woodland savanna on granite: Acacia, Bushwillow and Mopane; **Record Level:** type: PhysicalObject; institutionCode: ZMJU; basisOfRecord: PreservedSpecimen

#### Description

The identification was based on Hacker (2016).

#### Distribution

**Figure 7.**
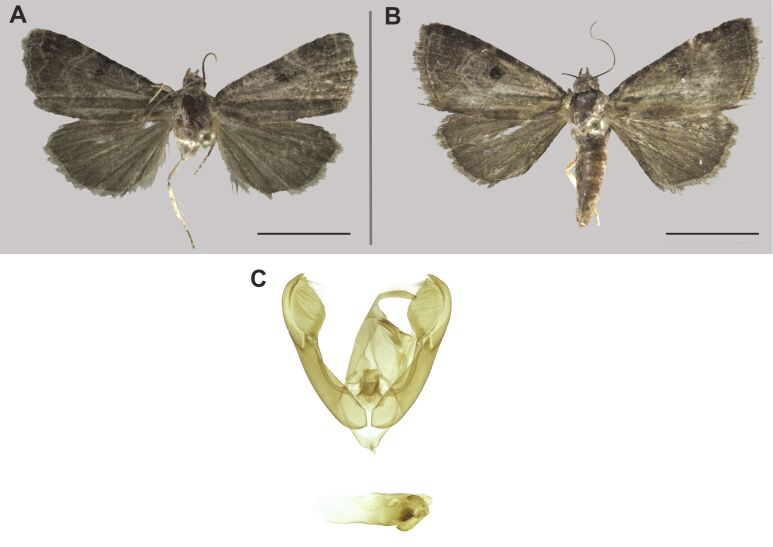
*Ozarbaberioi* Hacker, 2016, South Africa, Kruger National Park; **A** male; **B** female; **C** male genitalia. The scale represents 5 mm.

We report this species for the first time in South Africa. So far, this species has been known only from East Africa: the Somalian region (Ethiopia and Kenya) and the Zambezian region (Tanzania; Hacker 2016, De Prins and De Prins 2022). Therefore, our record extended its known distribution range for over 1,000 km southwards (Fig. 7).

### Ozarba bicolora

(Bethune-Baker, 1911)

6314595F-29A8-5D23-8D35-22A95EC81143

Noctuidae , Eustrotiinae

#### Materials

- **Type status:** Other material. **Occurrence:** individualCount: 1; sex: male; lifeStage: adult; **Taxon:** scientificName: Ozarbabicolora (Bethune-Baker, 1911); **Location:** country: South Africa; stateProvince: Limpopo; verbatimLocality: Kruger NP, Road S56, Mphongolo river, 1 km N of Ribye waterhole; verbatimElevation: 314 m; decimalLatitude: -22.991653; decimalLongitude: 31.270644; **Identification:** identifiedBy: Sylvain Delabye; dateIdentified: 2022; **Event:** eventDate: 25/02/2020; habitat: Perennial river; open savanna grassland with stunted mopane; **Record Level:** type: PhysicalObject; institutionCode: ZMJU; basisOfRecord: PreservedSpecimen
- **Type status:** Other material. **Occurrence:** individualCount: 1; sex: female; lifeStage: adult; **Taxon:** scientificName: Ozarbabicolora (Bethune-Baker, 1911); **Location:** country: South Africa; stateProvince: Limpopo; verbatimLocality: Kruger NP, Shisha West waterhole; verbatimElevation: 363 m; decimalLatitude: -22.827400; decimalLongitude: 31.216057; **Identification:** identifiedBy: Sylvain Delabye; dateIdentified: 2022; **Event:** eventDate: 25/02/2020; habitat: Seasonal river; open savanna grassland with stunted mopane; **Record Level:** type: PhysicalObject; institutionCode: ZMJU; basisOfRecord: PreservedSpecimen

#### Description

The identification was based on Hacker (2016).

#### Distribution

**Figure 8.**
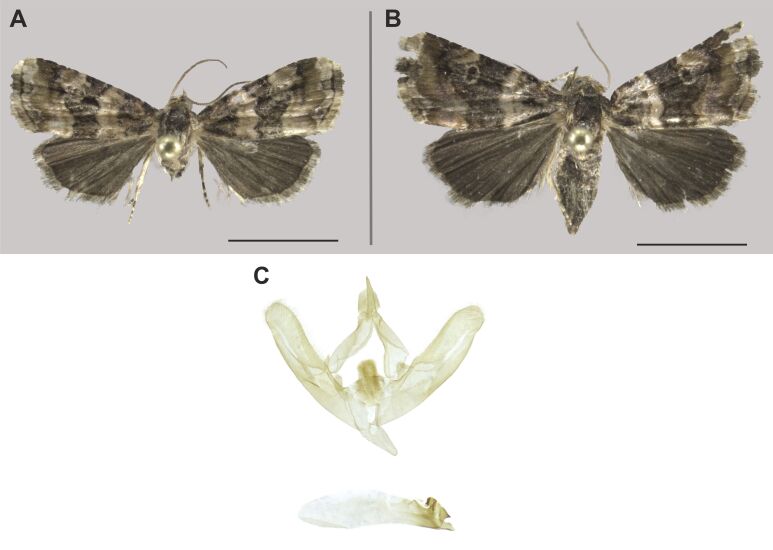
*Ozarbabicolora* (Bethune-Baker, 1911), South Africa, Kruger National Park; **A** male; **B** female; **C** male genitalia. The scale represents 5 mm.

We report this species for the first time in South Africa. This species was known only from Eastern Africa: the Somalian (Ethiopia and Kenya), Shaba (Uganda) and Zambezian (Tanzania) biogeographical regions (Hacker 2016, De Prins and De Prins 2022). Therefore, our record extended its known distribution for over 1,000 km southwards (Fig. 8).

### Ozarba exoplaga

Berio, 1940

CBD6AB5E-7AA5-5060-AC0F-01241EFC9B98

Noctuidae , Eustrotiinae

#### Materials

- **Type status:** Other material. **Occurrence:** individualCount: 1; sex: male; lifeStage: adult; **Taxon:** scientificName: Ozarbaexoplaga Berio, 1940; **Location:** country: South Africa; stateProvince: Limpopo; verbatimLocality: Kruger NP, Redrocks waterhole; verbatimElevation: 328 m; decimalLatitude: -23.172717; decimalLongitude: 31.299895; **Identification:** identifiedBy: Sylvain Delabye; dateIdentified: 2022; **Event:** eventDate: 24/02/2020; habitat: Perennial river; open savanna grassland with stunted mopane; **Record Level:** type: PhysicalObject; institutionCode: ZMJU; basisOfRecord: PreservedSpecimen
- **Type status:** Other material. **Occurrence:** individualCount: 1; sex: male; lifeStage: adult; **Taxon:** scientificName: Ozarbaexoplaga Berio, 1940; **Location:** country: South Africa; stateProvince: Mpumalanga; verbatimLocality: Kruger NP, N’wanetzi river, road S100, 2 km W of Nsasane waterhole; verbatimElevation: 228 m; decimalLatitude: -24.381219; decimalLongitude: 31.886093; **Identification:** identifiedBy: Sylvain Delabye; dateIdentified: 2022; **Event:** eventDate: 14/11/2018; habitat: Seasonal river; rugged veld: mixed thornveld and woodland savanna; **Record Level:** type: PhysicalObject; institutionCode: ZMJU; basisOfRecord: PreservedSpecimen
- **Type status:** Other material. **Occurrence:** individualCount: 3; sex: males; lifeStage: adult; **Taxon:** scientificName: Ozarbaexoplaga Berio, 1940; **Location:** country: South Africa; stateProvince: Limpopo; verbatimLocality: Kruger NP, Tsendze river, 2 km SE of Frazerus waterhole; verbatimElevation: 352 m; decimalLatitude: -23.495737; decimalLongitude: 31.325834; **Identification:** identifiedBy: Sylvain Delabye; dateIdentified: 2022; **Event:** eventDate: 28/02/2020; habitat: Seasonal river; mopani dominated woodland savanna on granite: Acacia, Bushwillow and Mopane; **Record Level:** type: PhysicalObject; institutionCode: IECA; basisOfRecord: PreservedSpecimen
- **Type status:** Other material. **Occurrence:** individualCount: 1; sex: male; lifeStage: adult; **Taxon:** scientificName: Ozarbaexoplaga Berio, 1940; **Location:** country: South Africa; stateProvince: Mpumalanga; verbatimLocality: Kruger NP, N’waswitsontso river, road S125, 1 km NW of Mhisanamond waterhole; verbatimElevation: 329 m; decimalLatitude: -24.600415; decimalLongitude: 31.687177; **Identification:** identifiedBy: Sylvain Delabye; dateIdentified: 2022; **Event:** eventDate: 16/11/2018; habitat: Perennial river; mixed thorn and Marula woodland savanna on granite: Bushwillow, Acacia and Marula; **Record Level:** type: PhysicalObject; institutionCode: IECA; basisOfRecord: PreservedSpecimen
- **Type status:** Other material. **Occurrence:** individualCount: 1; sex: male; lifeStage: adult; **Taxon:** scientificName: Ozarbaexoplaga Berio, 1940; **Location:** country: South Africa; stateProvince: Mpumalanga; verbatimLocality: Kruger NP, Road H1-3, 500m W of Marheya waterhole; verbatimElevation: 333 m; decimalLatitude: -24.565596; decimalLongitude: 31.774760; **Identification:** identifiedBy: Sylvain Delabye; dateIdentified: 2022; **Event:** eventDate: 01/03/2019; habitat: Crest; wooded savanna on shale: Bushwillow, Leadwood, Marula, Knobthorn trees, and thorn thickets; **Record Level:** type: PhysicalObject; institutionCode: IECA; basisOfRecord: PreservedSpecimen

#### Description

The identification was based on Hacker (2016).

#### Distribution

**Figure 9.**
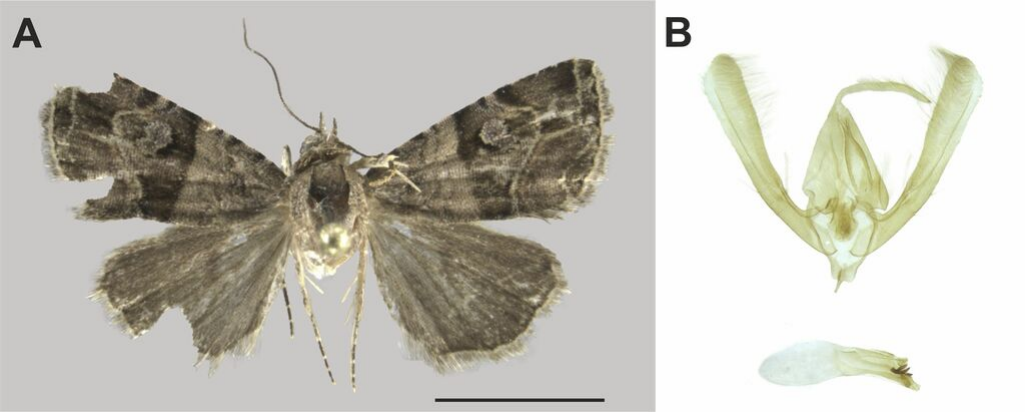
*Ozarbaexoplaga* Berio, 1940, South Africa, Kruger National Park; **A** male; **B** male genitalia. The scale represents 5 mm.

We report this species for the first time in South Africa. It was known from several countries in equatorial Africa (including Tanzania in the Zambezian biogeographic region) and Madagascar (Hacker 2016, De Prins and De Prins 2022). Therefore, the reported distribution in KNP extended the species’ known distribution on the continent for over 1,000 km southwards (Fig. 9).

### Ozarba gaedei

Berio, 1940

62126F33-DD5B-5E9C-A59E-4E28B3C4EDC1

Noctuidae , Eustrotiinae

#### Materials

- **Type status:** Other material. **Occurrence:** individualCount: 1; sex: male; lifeStage: adult; **Taxon:** scientificName: Ozarbagaedei Berio, 1940; **Location:** country: South Africa; stateProvince: Limpopo; verbatimLocality: Kruger NP, Nyawutsi waterhole; verbatimElevation: 288 m; decimalLatitude: -23.294739; decimalLongitude: 31.529902; **Identification:** identifiedBy: Sylvain Delabye; dateIdentified: 2022; **Event:** eventDate: 26/02/2020; habitat: Seasonal river; Lebombo - low, arid rhyolite hills: Acacia and thorn thickets; **Record Level:** type: PhysicalObject; institutionCode: ZMJU; basisOfRecord: PreservedSpecimen
- **Type status:** Other material. **Occurrence:** individualCount: 1; sex: male; lifeStage: adult; **Taxon:** scientificName: Ozarbagaedei Berio, 1940; **Location:** country: South Africa; stateProvince: Mpumalanga; verbatimLocality: Kruger NP, N’waswitsontso river, road S125, 1 km NW of Mhisanamond waterhole; verbatimElevation: 329 m; decimalLatitude: -24.600415; decimalLongitude: 31.687177; **Identification:** identifiedBy: Sylvain Delabye; dateIdentified: 2022; **Event:** eventDate: 03/03/2019; habitat: Perennial river; mixed thorn and Marula woodland savanna on granite: Bushwillow, Acacia and Marula; **Record Level:** type: PhysicalObject; institutionCode: ZMJU; basisOfRecord: PreservedSpecimen
- **Type status:** Other material. **Occurrence:** individualCount: 2; sex: males; lifeStage: adult; **Taxon:** scientificName: Ozarbagaedei Berio, 1940; **Location:** country: South Africa; stateProvince: Limpopo; verbatimLocality: Kruger NP, Nkokhodzi river, road H1-6; verbatimElevation: 358 m; decimalLatitude: -23.239622; decimalLongitude: 31.312995; **Identification:** identifiedBy: Sylvain Delabye; dateIdentified: 2022; **Event:** eventDate: 24/02/2020; habitat: Seasonal river; mopani dominated woodland savanna on granite: Acacia, Bushwillow and Mopane; **Record Level:** type: PhysicalObject; institutionCode: ZMJU; basisOfRecord: PreservedSpecimen
- **Type status:** Other material. **Occurrence:** individualCount: 1; sex: male; lifeStage: adult; **Taxon:** scientificName: Ozarbagaedei Berio, 1940; **Location:** country: South Africa; stateProvince: Limpopo; verbatimLocality: Kruger NP, Tinyarhini waterhole; verbatimElevation: 280 m; decimalLatitude: -23.151872; decimalLongitude: 31.475738; **Identification:** identifiedBy: Sylvain Delabye; dateIdentified: 2022; **Event:** eventDate: 28/11/2019; habitat: Open savanna grassland with stunted mopane; **Record Level:** type: PhysicalObject; institutionCode: IECA; basisOfRecord: PreservedSpecimen
- **Type status:** Other material. **Occurrence:** individualCount: 1; lifeStage: adult; **Taxon:** scientificName: Ozarbagaedei Berio, 1940; **Location:** country: South Africa; stateProvince: Mpumalanga; verbatimLocality: Kruger NP, Crocodile river, road S25, 11 km W of Crocodile Bridge camp; verbatimElevation: 195 m; decimalLatitude: -25.333097; decimalLongitude: 31.814744; **Identification:** identifiedBy: Sylvain Delabye; dateIdentified: 2022; **Event:** eventDate: 11/11/2018; habitat: Perennial river; mixed woodland savanna and thorn thickets: Tamboti, Acacia, Marula and Bushwillow; **Record Level:** type: PhysicalObject; institutionCode: IECA; basisOfRecord: PreservedSpecimen
- **Type status:** Other material. **Occurrence:** individualCount: 1; lifeStage: adult; **Taxon:** scientificName: Ozarbagaedei Berio, 1940; **Location:** country: South Africa; stateProvince: Limpopo; verbatimLocality: Kruger NP, Road S133, 6 km S of Jumbo waterhole; verbatimElevation: 365 m; decimalLatitude: -23.844016; decimalLongitude: 31.365157; **Identification:** identifiedBy: Sylvain Delabye; dateIdentified: 2022; **Event:** eventDate: 19/02/2020; habitat: Crest; mixed woodland savanna with sweet grazing: Knobthorn and Marula; **Record Level:** type: PhysicalObject; institutionCode: IECA; basisOfRecord: PreservedSpecimen
- **Type status:** Other material. **Occurrence:** individualCount: 1; lifeStage: adult; **Taxon:** scientificName: Ozarbagaedei Berio, 1940; **Location:** country: South Africa; stateProvince: Mpumalanga; verbatimLocality: Kruger NP, Crocodile river, Crocodile Bridge; verbatimElevation: 183 m; decimalLatitude: -25.363011; decimalLongitude: 31.891832; **Identification:** identifiedBy: Sylvain Delabye; dateIdentified: 2022; **Event:** eventDate: 07/03/2019; habitat: Perennial river; open savanna grassland on basalt; **Record Level:** type: PhysicalObject; institutionCode: IECA; basisOfRecord: PreservedSpecimen
- **Type status:** Other material. **Occurrence:** individualCount: 1; lifeStage: adult; **Taxon:** scientificName: Ozarbagaedei Berio, 1940; **Location:** country: South Africa; stateProvince: Mpumalanga; verbatimLocality: Kruger NP, N’waswitsontso river, road S125, 1 km NW of Mhisanamond waterhole; verbatimElevation: 325 m; decimalLatitude: -24.757101; decimalLongitude: 31.698319; **Identification:** identifiedBy: Sylvain Delabye; dateIdentified: 2022; **Event:** eventDate: 03/03/2019; habitat: Seasonal river; mixed thorn and Marula woodland savanna on granite: Bushwillow, Acacia and Marula; **Record Level:** type: PhysicalObject; institutionCode: IECA; basisOfRecord: PreservedSpecimen
- **Type status:** Other material. **Occurrence:** individualCount: 1; sex: female; lifeStage: adult; **Taxon:** scientificName: Ozarbagaedei Berio, 1940; **Location:** country: South Africa; stateProvince: Mpumalanga; verbatimLocality: Kruger NP, N’waswitshaka river, 4 km E of N’waswitshaka waterhole; verbatimElevation: 287 m; decimalLatitude: -25.026541; decimalLongitude: 31.549340; **Identification:** identifiedBy: Sylvain Delabye; dateIdentified: 2022; **Event:** eventDate: 27/02/2019; habitat: Seasonal river; mixed woodland savanna and thorn thickets: Tamboti, Acacia, Marula and Bushwillows; **Record Level:** type: PhysicalObject; institutionCode: IECA; basisOfRecord: PreservedSpecimen
- **Type status:** Other material. **Occurrence:** individualCount: 1; lifeStage: adult; **Taxon:** scientificName: Ozarbagaedei Berio, 1940; **Location:** country: South Africa; stateProvince: Limpopo; verbatimLocality: Kruger NP, Road S56, Mphongolo river, 1 km N of Ribye waterhole; verbatimElevation: 314 m; decimalLatitude: -22.991653; decimalLongitude: 31.270644; **Identification:** identifiedBy: Sylvain Delabye; dateIdentified: 2022; **Event:** eventDate: 29/11/2019; habitat: Perennial river; open savanna grassland with stunted mopane; **Record Level:** type: PhysicalObject; institutionCode: IECA; basisOfRecord: PreservedSpecimen
- **Type status:** Other material. **Occurrence:** individualCount: 1; lifeStage: adult; **Taxon:** scientificName: Ozarbagaedei Berio, 1940; **Location:** country: South Africa; stateProvince: Limpopo; verbatimLocality: Kruger NP, Road S47, Nwanedzi river; verbatimElevation: 278 m; decimalLatitude: -23.793044; decimalLongitude: 31.479490; **Identification:** identifiedBy: Sylvain Delabye; dateIdentified: 2022; **Event:** eventDate: 21/02/2020; habitat: Seasonal river; mopani dominated woodland savanna on granite: Acacia, Bushwillow and Mopane; **Record Level:** type: PhysicalObject; institutionCode: IECA; basisOfRecord: PreservedSpecimen

#### Description

The identification was based on Hacker (2016).

#### Distribution

**Figure 10.**
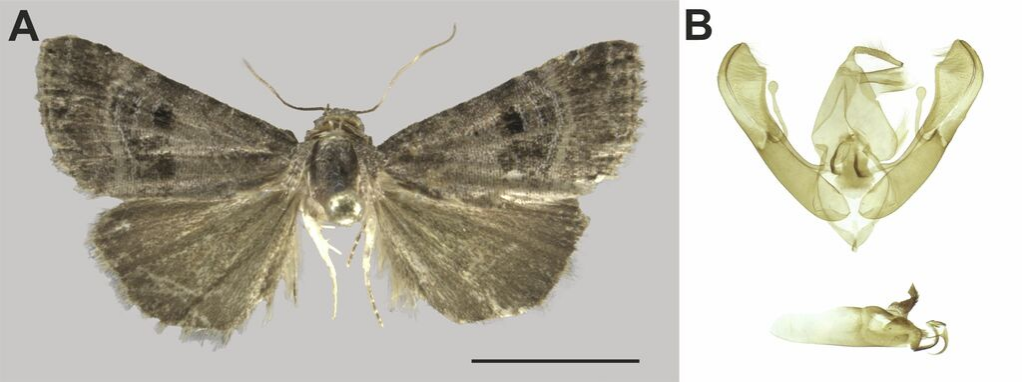
*Ozarbagaedei* Berio, 1940, South Africa, Kruger National Park; **A** male; **B** male genitalia. The scale represents 5 mm.

We report this species for the first time in South Africa. Previously, it was considered as endemic to Namibia (Hacker 2016, De Prins and De Prins 2022). Our records extended its distribution range for almost 2,000 km eastwards, into the Zambezian biogeographical region (Fig. 10).

### Ozarba hemileuca

Wiltshire, 1982

1C6E4112-97A6-5673-B937-06EDCA4505A4

Noctuidae , Eustrotiinae

#### Materials

- **Type status:** Other material. **Occurrence:** individualCount: 1; sex: male; lifeStage: adult; **Taxon:** scientificName: Ozarbahemileuca Wiltshire, 1982; **Location:** country: South Africa; stateProvince: Limpopo; verbatimLocality: Kruger NP, Letaba river, 10 km E of Letaba camp; verbatimElevation: 217 m; decimalLatitude: -23.844905; decimalLongitude: 31.641514; **Identification:** identifiedBy: Sylvain Delabye; dateIdentified: 2022; **Event:** eventDate: 01/12/2019; habitat: Perennial river; open savanna grassland with stunted mopane; **Record Level:** type: PhysicalObject; institutionCode: ZMJU; basisOfRecord: PreservedSpecimen
- **Type status:** Other material. **Occurrence:** individualCount: 1; sex: female; lifeStage: adult; **Taxon:** scientificName: Ozarbahemileuca Wiltshire, 1982; **Location:** country: South Africa; stateProvince: Limpopo; verbatimLocality: Kruger NP, Nkokhodzi river, road H1-6; verbatimElevation: 358 m; decimalLatitude: -23.239622; decimalLongitude: 31.312995; **Identification:** identifiedBy: Sylvain Delabye; dateIdentified: 2022; **Event:** eventDate: 24/02/2020; habitat: Seasonal river; mopani dominated woodland savanna on granite: Acacia, Bushwillow and Mopane; **Record Level:** type: PhysicalObject; institutionCode: ZMJU; basisOfRecord: PreservedSpecimen
- **Type status:** Other material. **Occurrence:** individualCount: 1; sex: female; lifeStage: adult; **Taxon:** scientificName: Ozarbahemileuca Wiltshire, 1982; **Location:** country: South Africa; stateProvince: Limpopo; verbatimLocality: Kruger NP, Shingwedzi river, Historical Site 50; verbatimElevation: 266 m; decimalLatitude: -23.220380; decimalLongitude: 31.552017; **Identification:** identifiedBy: Sylvain Delabye; dateIdentified: 2022; **Event:** eventDate: 26/02/2020; habitat: Perennial river; Lebombo - low, arid rhyolite hills: Acacia and thorn thickets; **Record Level:** type: PhysicalObject; institutionCode: ZMJU; basisOfRecord: PreservedSpecimen
- **Type status:** Other material. **Occurrence:** individualCount: 1; sex: male; lifeStage: adult; **Taxon:** scientificName: Ozarbahemileuca Wiltshire, 1982; **Location:** country: South Africa; stateProvince: Mpumalanga; verbatimLocality: Kruger NP, 1 km NW of Balule camp; verbatimElevation: 269 m; decimalLatitude: -24.053562; decimalLongitude: 31.722598; **Identification:** identifiedBy: Sylvain Delabye; dateIdentified: 2022; **Event:** eventDate: 20/11/2019; habitat: Perennial river; open savanna grassland with stunted mopane; **Record Level:** type: PhysicalObject; institutionCode: ZMJU; basisOfRecord: PreservedSpecimen
- **Type status:** Other material. **Occurrence:** individualCount: 2; sex: male; lifeStage: adult; **Taxon:** scientificName: Ozarbahemileuca Wiltshire, 1982; **Location:** country: South Africa; stateProvince: Mpumalanga; verbatimLocality: Kruger NP, N’wanetzi river, road S100, 2 km W of Nsasane waterhole; verbatimElevation: 228 m; decimalLatitude: -24.381219; decimalLongitude: 31.886093; **Identification:** identifiedBy: Sylvain Delabye; dateIdentified: 2022; **Event:** eventDate: 02/03/2019; habitat: Seasonal river; rugged veld: mixed thornveld and woodland savanna; **Record Level:** type: PhysicalObject; institutionCode: ZMJU; basisOfRecord: PreservedSpecimen
- **Type status:** Other material. **Occurrence:** individualCount: 1; sex: male; lifeStage: adult; **Taxon:** scientificName: Ozarbahemileuca Wiltshire, 1982; **Location:** country: South Africa; stateProvince: Limpopo; verbatimLocality: Kruger NP, Road S47, Nwanedzi river; verbatimElevation: 278 m; decimalLatitude: -23.793044; decimalLongitude: 31.479490; **Identification:** identifiedBy: Sylvain Delabye; dateIdentified: 2022; **Event:** eventDate: 02/12/2019; habitat: Seasonal river; mopani dominated woodland savanna on granite: Acacia, Bushwillow and Mopane; **Record Level:** type: PhysicalObject; institutionCode: ZMJU; basisOfRecord: PreservedSpecimen
- **Type status:** Other material. **Occurrence:** individualCount: 1; sex: female; lifeStage: adult; **Taxon:** scientificName: Ozarbahemileuca Wiltshire, 1982; **Location:** country: South Africa; stateProvince: Limpopo; verbatimLocality: Kruger NP, Road S47, Nwanedzi river; verbatimElevation: 278 m; decimalLatitude: -23.793044; decimalLongitude: 31.479490; **Identification:** identifiedBy: Sylvain Delabye; dateIdentified: 2022; **Event:** eventDate: 21/02/2020; habitat: Seasonal river; mopani dominated woodlandsavanna on granite: Acacia, Bushwillow and Mopane; **Record Level:** type: PhysicalObject; institutionCode: ZMJU; basisOfRecord: PreservedSpecimen

#### Description

The identification was based on Hacker (2016).

#### Distribution

**Figure 11.**
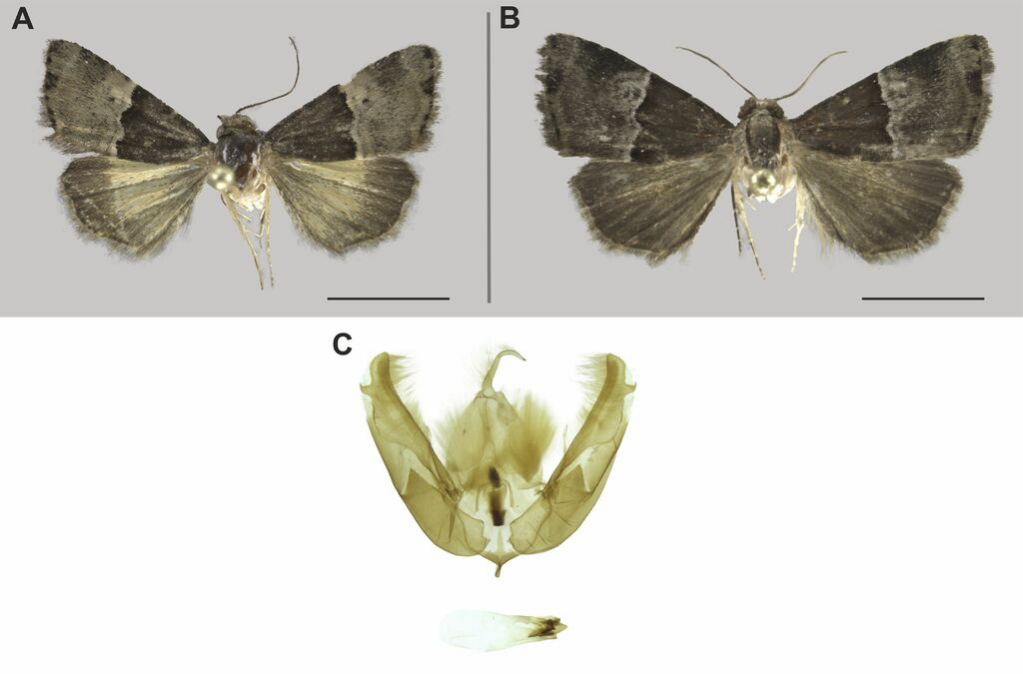
*Ozarbahemileuca* Wiltshire, 1982, South Africa, Kruger National Park; **A** male; **B** female; **C** male genitalia. The scale represents 5 mm.

We report this species for the first time in South Africa. This species was known from the Somalian (Ethiopia, Somalia and Kenya) and Zambezian (Tanzania) biogeographic regions (Hacker 2016, De Prins and De Prins 2022). Our records from South Africa thus extended its known distribution for over 1,000 km southwards (Fig. 11).

### Ozarba limbata

(Butler, 1898)

48382CDA-4166-5777-90AD-CD4ACB80FACF

Noctuidae , Eustrotiinae

#### Materials

- **Type status:** Other material. **Occurrence:** individualCount: 3; sex: males; lifeStage: adult; **Taxon:** scientificName: Ozarbalimbata (Butler, 1898); **Location:** country: South Africa; stateProvince: Limpopo; verbatimLocality: Kruger NP, Redrocks waterhole; verbatimElevation: 328 m; decimalLatitude: -23.172717; decimalLongitude: 31.299895; **Identification:** identifiedBy: Sylvain Delabye; dateIdentified: 2022; **Event:** eventDate: 26/11/2019; habitat: Perennial river; open savanna grassland with stunted mopane; **Record Level:** type: PhysicalObject; institutionCode: IECA; basisOfRecord: PreservedSpecimen
- **Type status:** Other material. **Occurrence:** individualCount: 1; sex: male; lifeStage: adult; **Taxon:** scientificName: Ozarbalimbata (Butler, 1898); **Location:** country: South Africa; stateProvince: Limpopo; verbatimLocality: Kruger NP, Tinyarhini waterhole; verbatimElevation: 280 m; decimalLatitude: -23.151872; decimalLongitude: 31.475738; **Identification:** identifiedBy: Sylvain Delabye; dateIdentified: 2022; **Event:** eventDate: 28/11/2019; habitat: Open savanna grassland with stunted mopane; **Record Level:** type: PhysicalObject; institutionCode: IECA; basisOfRecord: PreservedSpecimen
- **Type status:** Other material. **Occurrence:** individualCount: 1; sex: male; lifeStage: adult; **Taxon:** scientificName: Ozarbalimbata (Butler, 1898); **Location:** country: South Africa; stateProvince: Limpopo; verbatimLocality: Kruger NP, Road S47, Nwanedzi river; verbatimElevation: 278 m; decimalLatitude: -23.793044; decimalLongitude: 31.479490; **Identification:** identifiedBy: Sylvain Delabye; dateIdentified: 2022; **Event:** eventDate: 02/12/2019; habitat: Seasonal river; mopani dominated woodland savanna on granite: Acacia, Bushwillow and Mopane; **Record Level:** type: PhysicalObject; institutionCode: IECA; basisOfRecord: PreservedSpecimen
- **Type status:** Other material. **Occurrence:** individualCount: 1; sex: male; lifeStage: adult; **Taxon:** scientificName: Ozarbalimbata (Butler, 1898); **Location:** country: South Africa; stateProvince: Limpopo; verbatimLocality: Kruger NP, Nkokhodzi river, road H1-6; verbatimElevation: 358 m; decimalLatitude: -23.239622; decimalLongitude: 31.312995; **Identification:** identifiedBy: Sylvain Delabye; dateIdentified: 2022; **Event:** eventDate: 26/11/2019; habitat: Seasonal river; mopani dominated woodland savanna on granite: Acacia, Bushwillow and Mopane; **Record Level:** type: PhysicalObject; institutionCode: IECA; basisOfRecord: PreservedSpecimen

#### Description

The identification was based on Hacker (2016).

#### Distribution

**Figure 12.**
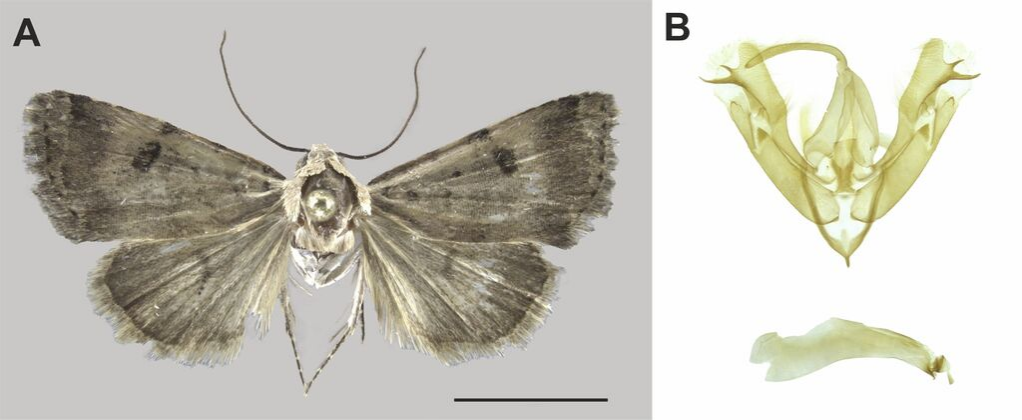
*Ozarbalimbata* (Butler, 1898), South Africa, Kruger National Park; **A** male; **B** male genitalia. The scale represents 5 mm.

We report this species for the first time in South Africa. It was previously known from several countries in Eastern Africa (including Tanzania in the Zambezian biogeographic region; (Hacker 2016, De Prins and De Prins 2022). Our South African record thus extended its distribution for over 1,000 km southwards (Fig. 12).

### Ozarba negrottoi

Berio, 1940

41A77824-6CC0-5BBE-BAEF-1886C87425AF

Noctuidae , Eustrotiinae

#### Materials

- **Type status:** Other material. **Occurrence:** individualCount: 1; sex: male; lifeStage: adult; **Taxon:** scientificName: Ozarbanegrottoi Berio, 1940; **Location:** country: South Africa; stateProvince: Limpopo; verbatimLocality: Kruger NP, Tsendze river, 2 km SE of Frazerus waterhole; verbatimElevation: 352 m; decimalLatitude: -23.495737; decimalLongitude: 31.325834; **Identification:** identifiedBy: Sylvain Delabye; dateIdentified: 2022; **Event:** eventDate: 30/11/2019; habitat: Seasonal river; mopani dominated woodland savanna on granite: Acacia, Bushwillow and Mopane; **Record Level:** type: PhysicalObject; institutionCode: ZMJU; basisOfRecord: PreservedSpecimen
- **Type status:** Other material. **Occurrence:** individualCount: 1; sex: female; lifeStage: adult; **Taxon:** scientificName: Ozarbanegrottoi Berio, 1940; **Location:** country: South Africa; stateProvince: Limpopo; verbatimLocality: Kruger NP, Road S56, Mphongolo river, 1 km N of Ribye waterhole; verbatimElevation: 314 m; decimalLatitude: -22.991653; decimalLongitude: 31.270644; **Identification:** identifiedBy: Sylvain Delabye; dateIdentified: 2022; **Event:** eventDate: 29/11/2019; habitat: Perennial river; open savanna grassland with stunted mopane; **Record Level:** type: PhysicalObject; institutionCode: ZMJU; basisOfRecord: PreservedSpecimen
- **Type status:** Other material. **Occurrence:** individualCount: 1; sex: male; lifeStage: adult; **Taxon:** scientificName: Ozarbanegrottoi Berio, 1940; **Location:** country: South Africa; stateProvince: Limpopo; verbatimLocality: Kruger NP, Letaba river, 6 km N of Letaba camp; verbatimElevation: 245 m; decimalLatitude: -23.811079; decimalLongitude: 31.580949; **Identification:** identifiedBy: Sylvain Delabye; dateIdentified: 2022; **Event:** eventDate: 25/11/2019; habitat: Perennial river; open savanna grassland with stunted mopane; **Record Level:** type: PhysicalObject; institutionCode: ZMJU; basisOfRecord: PreservedSpecimen

#### Description

The identification was based on Hacker (2016).

#### Distribution

**Figure 13.**
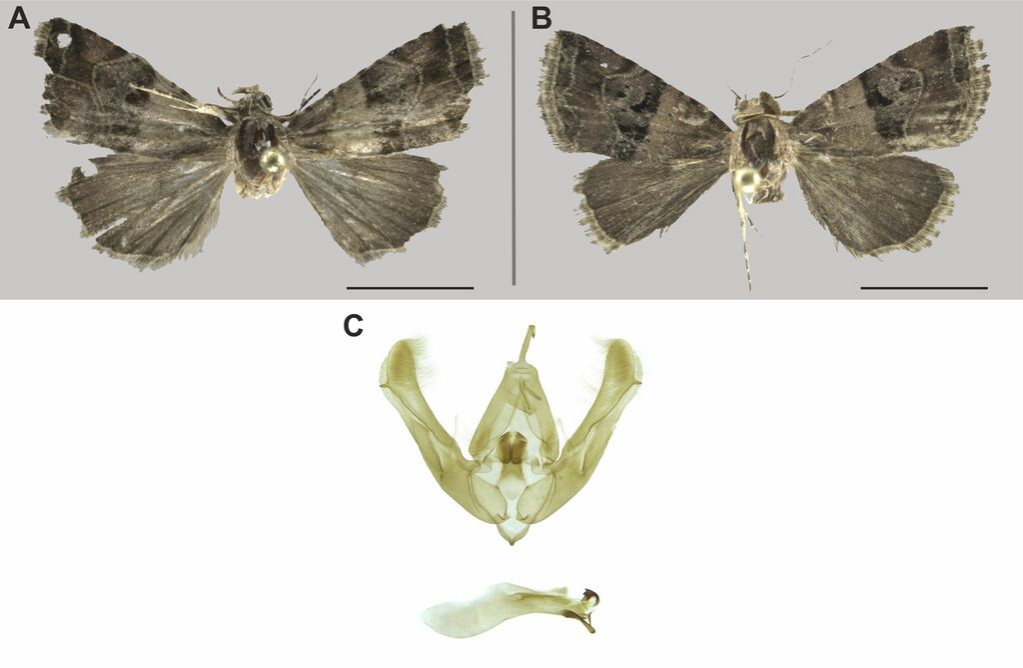
*Ozarbanegrottoi* Berio, 1940, South Africa, Kruger National Park; **A** male; **B** female; **C** male genitalia. The scale represents 5 mm.

The three reported individuals represent the first record of *O.negrottoi* in South Africa. This species was previously known only from the Somalian (Somalia) and Zambezian (Tanzania) regions (Hacker 2016, De Prins and De Prins 2022). We extended its known distribution for over 1,000 km southwards (Fig. 13).

### Ozarba persinua

Berio, 1940

AB91E3CB-3CB3-5CDD-979B-B0BCDCAC91E5

Noctuidae , Eustrotiinae

#### Materials

- **Type status:** Other material. **Occurrence:** individualCount: 1; sex: male; lifeStage: adult; **Taxon:** scientificName: Ozarbapersinua Berio, 1940; **Location:** country: South Africa; stateProvince: Limpopo; verbatimLocality: Kruger NP, Road H9, close to Misumani river; verbatimElevation: 379 m; decimalLatitude: -23.930505; decimalLongitude: 31.398723; **Identification:** identifiedBy: Sylvain Delabye; dateIdentified: 2022; **Event:** eventDate: 21/02/2020; habitat: Crest; mixed woodland savanna with sweet grazing: Knobthorn and Marula; **Record Level:** type: PhysicalObject; institutionCode: ZMJU; basisOfRecord: PreservedSpecimen
- **Type status:** Other material. **Occurrence:** individualCount: 1; sex: female; lifeStage: adult; **Taxon:** scientificName: Ozarbapersinua Berio, 1940; **Location:** country: South Africa; stateProvince: Limpopo; verbatimLocality: Kruger NP, Road S47, Nwanedzi river; verbatimElevation: 278 m; decimalLatitude: -23.793044; decimalLongitude: 31.479490; **Identification:** identifiedBy: Sylvain Delabye; dateIdentified: 2022; **Event:** eventDate: 21/02/2020; habitat: Seasonal river; mopani dominated woodland savanna on granite: Acacia, Bushwillow and Mopane; **Record Level:** type: PhysicalObject; institutionCode: ZMJU; basisOfRecord: PreservedSpecimen
- **Type status:** Other material. **Occurrence:** individualCount: 1; sex: male; lifeStage: adult; **Taxon:** scientificName: Ozarbapersinua Berio, 1940; **Location:** country: South Africa; stateProvince: Limpopo; verbatimLocality: Kruger NP, Tsendze river, 2 km SE of Frazerus waterhole; verbatimElevation: 352 m; decimalLatitude: -23.495737; decimalLongitude: 31.325834; **Identification:** identifiedBy: Sylvain Delabye; dateIdentified: 2022; **Event:** eventDate: 30/11/2019; habitat: Seasonal river; mopani dominated woodland savanna on granite: Acacia, Bushwillow and Mopane; **Record Level:** type: PhysicalObject; institutionCode: ZMJU; basisOfRecord: PreservedSpecimen
- **Type status:** Other material. **Occurrence:** individualCount: 2; lifeStage: adult; **Taxon:** scientificName: Ozarbapersinua Berio, 1940; **Location:** country: South Africa; stateProvince: Limpopo; verbatimLocality: Kruger NP, Road S56, Mphongolo river, 1 km N of Ribye waterhole; verbatimElevation: 314 m; decimalLatitude: -22.991653; decimalLongitude: 31.270644; **Identification:** identifiedBy: Sylvain Delabye; dateIdentified: 2022; **Event:** eventDate: 29/11/2019; habitat: Perennial river; open savanna grassland with stunted mopane; **Record Level:** type: PhysicalObject; institutionCode: IECA; basisOfRecord: PreservedSpecimen
- **Type status:** Other material. **Occurrence:** individualCount: 5; lifeStage: adult; **Taxon:** scientificName: Ozarbapersinua Berio, 1940; **Location:** country: South Africa; stateProvince: Mpumalanga; verbatimLocality: Kruger NP, Crocodile river, Crocodile Bridge; verbatimElevation: 183 m; decimalLatitude: -25.363011; decimalLongitude: 31.891832; **Identification:** identifiedBy: Sylvain Delabye; dateIdentified: 2022; **Event:** eventDate: 07/03/2019; habitat: Perennial river; open savanna grassland on basalt; **Record Level:** type: PhysicalObject; institutionCode: IECA; basisOfRecord: PreservedSpecimen
- **Type status:** Other material. **Occurrence:** individualCount: 1; lifeStage: adult; **Taxon:** scientificName: Ozarbapersinua Berio, 1940; **Location:** country: South Africa; stateProvince: Mpumalanga; verbatimLocality: Kruger NP, Vurhami river, road H4-2; verbatimElevation: 219 m; decimalLatitude: -25.246385; decimalLongitude: 31.847472; **Identification:** identifiedBy: Sylvain Delabye; dateIdentified: 2022; **Event:** eventDate: 07/03/2019; habitat: Season river; wooded savanna on shale: Bushwillow, Leadwood, Marula and Knobthorn trees, and thorn thickets; **Record Level:** type: PhysicalObject; institutionCode: IECA; basisOfRecord: PreservedSpecimen
- **Type status:** Other material. **Occurrence:** individualCount: 1; lifeStage: adult; **Taxon:** scientificName: Ozarbapersinua Berio, 1940; **Location:** country: South Africa; stateProvince: Mpumalanga; verbatimLocality: Kruger NP, N’watimhiri river, road S21, 1 km NE of Nhlotini waterhole; verbatimElevation: 283 m; decimalLatitude: -25.116682; decimalLongitude: 31.696896; **Identification:** identifiedBy: Sylvain Delabye; dateIdentified: 2022; **Event:** eventDate: 17/11/2018; habitat: Seasonal river; mixed woodland savanna and thorn thickets: Tamboti, Acacia, Marula and Bushwillows; **Record Level:** type: PhysicalObject; institutionCode: IECA; basisOfRecord: PreservedSpecimen
- **Type status:** Other material. **Occurrence:** individualCount: 1; lifeStage: adult; **Taxon:** scientificName: Ozarbapersinua Berio, 1940; **Location:** country: South Africa; stateProvince: Limpopo; verbatimLocality: Kruger NP, Letaba river, 10 km E of Letaba camp; verbatimElevation: 217 m; decimalLatitude: -23.844905; decimalLongitude: 31.641514; **Identification:** identifiedBy: Sylvain Delabye; dateIdentified: 2022; **Event:** eventDate: 01/12/2019; habitat: Perennial river; open savanna grassland with stunted mopane; **Record Level:** type: PhysicalObject; institutionCode: IECA; basisOfRecord: PreservedSpecimen
- **Type status:** Other material. **Occurrence:** individualCount: 1; lifeStage: adult; **Taxon:** scientificName: Ozarbapersinua Berio, 1940; **Location:** country: South Africa; stateProvince: Limpopo; verbatimLocality: Kruger NP, Stapelkop dam; verbatimElevation: 362 m; decimalLatitude: -23.594645; decimalLongitude: 31.254819; **Identification:** identifiedBy: Sylvain Delabye; dateIdentified: 2022; **Event:** eventDate: 30/11/2019; habitat: Perennial river; mopani dominated woodland savanna on granite: Acacia, Bushwillow and Mopane; **Record Level:** type: PhysicalObject; institutionCode: IECA; basisOfRecord: PreservedSpecimen
- **Type status:** Other material. **Occurrence:** individualCount: 7; lifeStage: adult; **Taxon:** scientificName: Ozarbapersinua Berio, 1940; **Location:** country: South Africa; stateProvince: Limpopo; verbatimLocality: Kruger NP, Redrocks waterhole; verbatimElevation: 328 m; decimalLatitude: -23.172717; decimalLongitude: 31.299895; **Identification:** identifiedBy: Sylvain Delabye; dateIdentified: 2022; **Event:** eventDate: 26/11/2019; habitat: Perennial river; open savanna grassland with stunted mopane; **Record Level:** type: PhysicalObject; institutionCode: IECA; basisOfRecord: PreservedSpecimen
- **Type status:** Other material. **Occurrence:** individualCount: 1; lifeStage: adult; **Taxon:** scientificName: Ozarbapersinua Berio, 1940; **Location:** country: South Africa; stateProvince: Limpopo; verbatimLocality: Kruger NP, Road H9, close to Misumani river; verbatimElevation: 379 m; decimalLatitude: -23.930505; decimalLongitude: 31.398723; **Identification:** identifiedBy: Sylvain Delabye; dateIdentified: 2022; **Event:** eventDate: 02/12/2019; habitat: Crest; mixed woodland savanna with sweet grazing: Knobthorn and Marula; **Record Level:** type: PhysicalObject; institutionCode: IECA; basisOfRecord: PreservedSpecimen
- **Type status:** Other material. **Occurrence:** individualCount: 1; lifeStage: adult; **Taxon:** scientificName: Ozarbapersinua Berio, 1940; **Location:** country: South Africa; stateProvince: Limpopo; verbatimLocality: Kruger NP, Road H1-6, 1 km SW of Olifantsbad pan; verbatimElevation: 426 m; decimalLatitude: -23.335829; decimalLongitude: 31.325420; **Identification:** identifiedBy: Sylvain Delabye; dateIdentified: 2022; **Event:** eventDate: 26/11/2019; habitat: Crest; mopani dominated woodland savanna on granite: Acacia, Bushwillow and Mopane; **Record Level:** type: PhysicalObject; institutionCode: IECA; basisOfRecord: PreservedSpecimen
- **Type status:** Other material. **Occurrence:** individualCount: 1; lifeStage: adult; **Taxon:** scientificName: Ozarbapersinua Berio, 1940; **Location:** country: South Africa; stateProvince: Mpumalanga; verbatimLocality: Kruger NP, N’waswitsontso river, road S86; verbatimElevation: 295 m; decimalLatitude: -24.678816; decimalLongitude: 31.791085; **Identification:** identifiedBy: Sylvain Delabye; dateIdentified: 2022; **Event:** eventDate: 01/03/2019; habitat: Seasonal river; mixed thorn and Marula woodland savanna on granite: Bushwillow, Acacia and Marula; **Record Level:** type: PhysicalObject; institutionCode: IECA; basisOfRecord: PreservedSpecimen
- **Type status:** Other material. **Occurrence:** individualCount: 1; lifeStage: adult; **Taxon:** scientificName: Ozarbapersinua Berio, 1940; **Location:** country: South Africa; stateProvince: Mpumalanga; verbatimLocality: Kruger NP, road S36, 5 km NE of Hlanguleni picnic site; verbatimElevation: 333 m; decimalLatitude: -24.681967; decimalLongitude: 31.666664; **Identification:** identifiedBy: Sylvain Delabye; dateIdentified: 2022; **Event:** eventDate: 03/03/2019; habitat: Crest; mixed thorn and Marula woodland savanna on granite: Bushwillow, Acacia and Marula; **Record Level:** type: PhysicalObject; institutionCode: IECA; basisOfRecord: PreservedSpecimen
- **Type status:** Other material. **Occurrence:** individualCount: 1; lifeStage: adult; **Taxon:** scientificName: Ozarbapersinua Berio, 1940; **Location:** country: South Africa; stateProvince: Mpumalanga; verbatimLocality: Kruger NP, Sabie river, Lookout on S79 road; verbatimElevation: 200 m; decimalLatitude: -25.064099; decimalLongitude: 31.825735; **Identification:** identifiedBy: Sylvain Delabye; dateIdentified: 2022; **Event:** eventDate: 24/02/2019; habitat: Perennial river; mixed woodland savanna and thorn thickets: Tamboti, Acacia, Marula and Bushwillow; **Record Level:** type: PhysicalObject; institutionCode: IECA; basisOfRecord: PreservedSpecimen
- **Type status:** Other material. **Occurrence:** individualCount: 1; lifeStage: adult; **Taxon:** scientificName: Ozarbapersinua Berio, 1940; **Location:** country: South Africa; stateProvince: Mpumalanga; verbatimLocality: Kruger NP, N’waswitsontso river, road S86; verbatimElevation: 295 m; decimalLatitude: -24.678816; decimalLongitude: 31.791085; **Identification:** identifiedBy: Sylvain Delabye; dateIdentified: 2022; **Event:** eventDate: 15/11/2018; habitat: Seasonal river; mixed thorn and Marula woodland savanna on granite: Bushwillow, Acacia and Marula; **Record Level:** type: PhysicalObject; institutionCode: IECA; basisOfRecord: PreservedSpecimen
- **Type status:** Other material. **Occurrence:** individualCount: 2; lifeStage: adult; **Taxon:** scientificName: Ozarbapersinua Berio, 1940; **Location:** country: South Africa; stateProvince: Limpopo; verbatimLocality: Kruger NP, Nyawutsi waterhole; verbatimElevation: 288 m; decimalLatitude: -23.294739; decimalLongitude: 31.529902; **Identification:** identifiedBy: Sylvain Delabye; dateIdentified: 2022; **Event:** eventDate: 27/11/2019; habitat: Seasonal river; Lebombo - low, arid rhyolite hills: Acacia and thorn thickets; **Record Level:** type: PhysicalObject; institutionCode: IECA; basisOfRecord: PreservedSpecimen
- **Type status:** Other material. **Occurrence:** individualCount: 1; lifeStage: adult; **Taxon:** scientificName: Ozarbapersinua Berio, 1940; **Location:** country: South Africa; stateProvince: Mpumalanga; verbatimLocality: Kruger NP, Sabie river, road H4-1, 7.5 km E fo Skukuza camp; verbatimElevation: 265 m; decimalLatitude: -24.973743; decimalLongitude: 31.661434; **Identification:** identifiedBy: Sylvain Delabye; dateIdentified: 2022; **Event:** eventDate: 06/11/2018; habitat: Perennial river; mixed woodland savanna and thorn thickets: Tamboti, Acacia, Marula and Bushwillow; **Record Level:** type: PhysicalObject; institutionCode: IECA; basisOfRecord: PreservedSpecimen
- **Type status:** Other material. **Occurrence:** individualCount: 2; lifeStage: adult; **Taxon:** scientificName: Ozarbapersinua Berio, 1940; **Location:** country: South Africa; stateProvince: Limpopo; verbatimLocality: Kruger NP, Letaba river, 6 km N of Letaba camp; verbatimElevation: 245 m; decimalLatitude: -23.811079; decimalLongitude: 31.580949; **Identification:** identifiedBy: Sylvain Delabye; dateIdentified: 2022; **Event:** eventDate: 25/11/2019; habitat: Perennial river; open savanna grassland with stunted mopane; **Record Level:** type: PhysicalObject; institutionCode: IECA; basisOfRecord: PreservedSpecimen
- **Type status:** Other material. **Occurrence:** individualCount: 1; lifeStage: adult; **Taxon:** scientificName: Ozarbapersinua Berio, 1940; **Location:** country: South Africa; stateProvince: Limpopo; verbatimLocality: Kruger NP, Road S47, Nwanedzi river; verbatimElevation: 278 m; decimalLatitude: -23.793044; decimalLongitude: 31.479490; **Identification:** identifiedBy: Sylvain Delabye; dateIdentified: 2022; **Event:** eventDate: 02/12/2019; habitat: Seasonal river; mopani dominated woodland savanna on granite: Acacia, Bushwillow and Mopane; **Record Level:** type: PhysicalObject; institutionCode: IECA; basisOfRecord: PreservedSpecimen
- **Type status:** Other material. **Occurrence:** individualCount: 1; lifeStage: adult; **Taxon:** scientificName: Ozarbapersinua Berio, 1940; **Location:** country: South Africa; stateProvince: Mpumalanga; verbatimLocality: Kruger NP, Crocodile river, road S114, 5 km N of Malelane gate; verbatimElevation: 300 m; decimalLatitude: -25.437282; decimalLongitude: 31.528655; **Identification:** identifiedBy: Sylvain Delabye; dateIdentified: 2022; **Event:** eventDate: 25/02/2019; habitat: Perennial river; mixed woodland savanna and thorn thickets: Tamboti, Acacia, Marula and Bushwillow; **Record Level:** type: PhysicalObject; institutionCode: IECA; basisOfRecord: PreservedSpecimen
- **Type status:** Other material. **Occurrence:** individualCount: 2; lifeStage: adult; **Taxon:** scientificName: Ozarbapersinua Berio, 1940; **Location:** country: South Africa; stateProvince: Limpopo; verbatimLocality: Kruger NP, Makhadzi waterhole; verbatimElevation: 285 m; decimalLatitude: -23.697166; decimalLongitude: 31.617761; **Identification:** identifiedBy: Sylvain Delabye; dateIdentified: 2022; **Event:** eventDate: 01/12/2019; habitat: Seasonal river; open savanna grassland with stunted mopane; **Record Level:** type: PhysicalObject; institutionCode: IECA; basisOfRecord: PreservedSpecimen
- **Type status:** Other material. **Occurrence:** individualCount: 1; lifeStage: adult; **Taxon:** scientificName: Ozarbapersinua Berio, 1940; **Location:** country: South Africa; stateProvince: Limpopo; verbatimLocality: Kruger NP, Nkokhodzi river, road H1-6; verbatimElevation: 358 m; decimalLatitude: -23.239622; decimalLongitude: 31.312995; **Identification:** identifiedBy: Sylvain Delabye; dateIdentified: 2022; **Event:** eventDate: 26/11/2019; habitat: Seasonal river; mopani dominated woodland savanna on granite: Acacia, Bushwillow and Mopane; **Record Level:** type: PhysicalObject; institutionCode: IECA; basisOfRecord: PreservedSpecimen
- **Type status:** Other material. **Occurrence:** individualCount: 1; lifeStage: adult; **Taxon:** scientificName: Ozarbapersinua Berio, 1940; **Location:** country: South Africa; stateProvince: Limpopo; verbatimLocality: Kruger NP, Tsendze river, 2 km SE of Frazerus waterhole; verbatimElevation: 352 m; decimalLatitude: -23.495737; decimalLongitude: 31.325834; **Identification:** identifiedBy: Sylvain Delabye; dateIdentified: 2022; **Event:** eventDate: 30/11/2019; habitat: Seasonal river; mopani dominated woodland savanna on granite: Acacia, Bushwillow and Mopane; **Record Level:** type: PhysicalObject; institutionCode: IECA; basisOfRecord: PreservedSpecimen

#### Description

The identification was based on Hacker (2016).

#### Distribution

**Figure 14.**
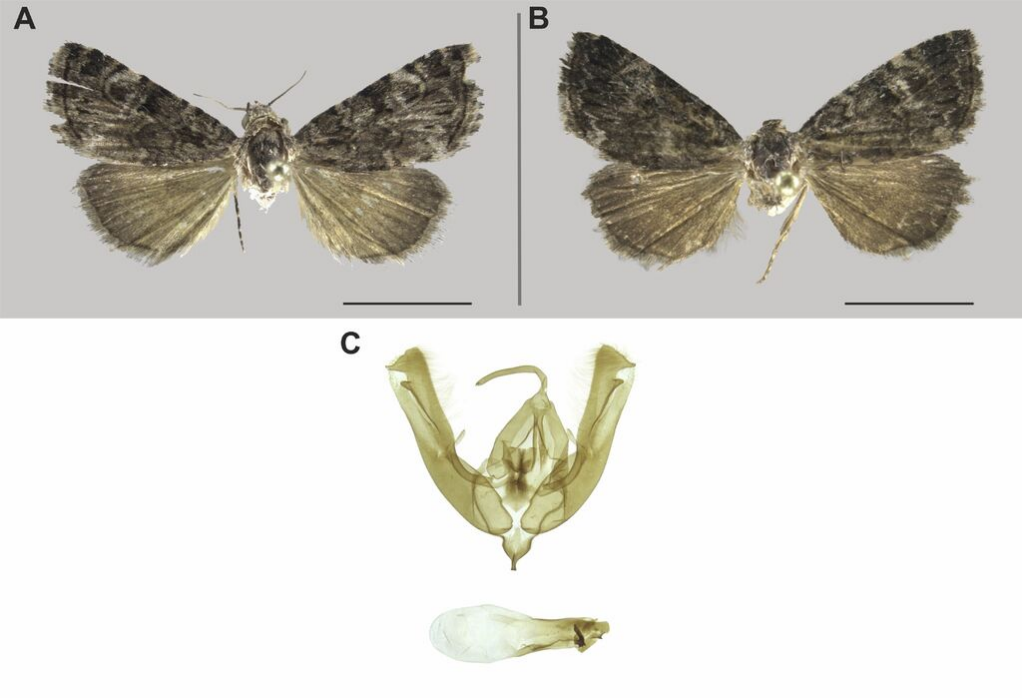
*Ozarbapersinua* Berio, 1940, South Africa, Kruger National Park; **A** male; **B** female; **C** male genitalia. The scale represents 5 mm.

This species was considered as endemic to Namibia (Hacker 2016, De Prins and De Prins 2022) and our specimens represent the first records of this species in South Africa. We therefore extended the species’ range distribution for almost 2,000 km eastwards, to the Zambezian biogeographic region (Fig. 14).

## Discussion

Altogether, we presented records of 13 species of moths newly reported to occur in South Africa. Two of these species (*O.gaedei* and *O.persinua*) were known to occur in Namibia (Southern African biogeographic region) only and our records extended their known distributions for over 2,000 km into the Zambezian biogeographic region. Although the remaining eleven species were previously recorded in the Zambezian biogeographic region (De Prins and De Prins 2022), the nearest known localities of eight of them were in Tanzania, i.e. over 1,000 km north of KNP. Only three reported species were already known from Zimbabwe, the country bordering KNP and their occurrence in South Africa could, therefore, be expected.

The new distributional records of moth species in KNP evidenced the poor knowledge on the lepidopteran fauna of the Afrotropical savannah ecosystems (Delabye et al. 2020, Mecenero et al. 2020). For most of the reported species, we extended the known distribution for over 1,000 km. As the ecosystems between KNP and the nearest reported localities of these species in Tanzania and/or Namibia include various types of savannahs (Mucina and Rutherford 2006, Delabye et al. 2020), we can assume that populations of at least some of these moths are being overlooked. This can be expected especially for the species which we report as relatively abundant in KNP (e.g. *E.accedens*, *O.atrisigna*, *O.persinua*). Therefore, we suggest an intensive survey of lepidopteran diversity in South Africa with results published even in small reports is crucial for improving our knowledge on the diversity of Afrotropical Lepidoptera.

## Supplementary Material

XML Treatment for Eublemma accedens aethiopica

XML Treatment for Eublemma savour

XML Treatment for Beriodesma determinata

XML Treatment for Heteropalpia exarata

XML Treatment for Ozarba atrisigna

XML Treatment for Ozarba berioi

XML Treatment for Ozarba bicolora

XML Treatment for Ozarba exoplaga

XML Treatment for Ozarba gaedei

XML Treatment for Ozarba hemileuca

XML Treatment for Ozarba limbata

XML Treatment for Ozarba negrottoi

XML Treatment for Ozarba persinua

## Figures and Tables

**Figure 1a.**
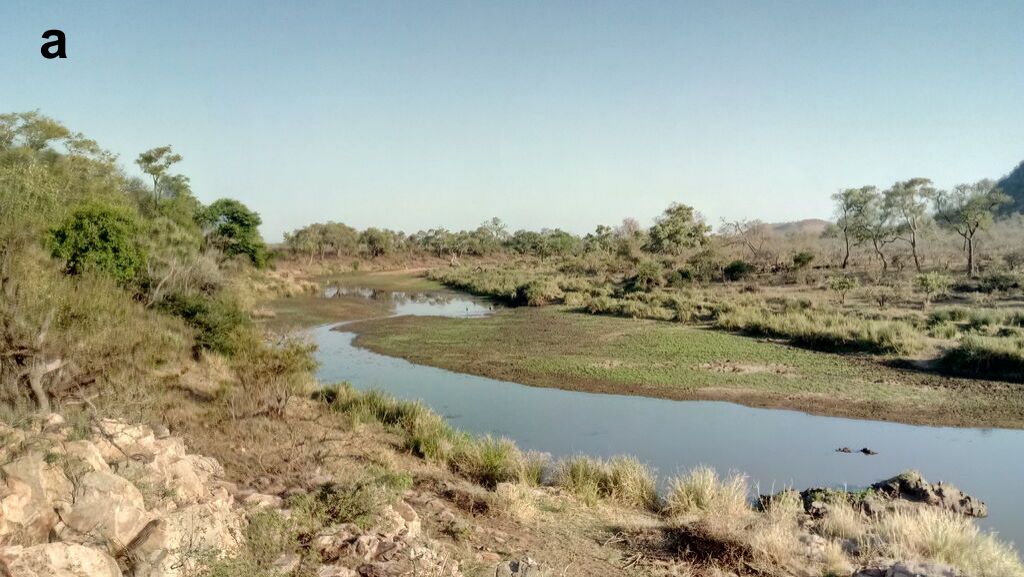
Mixed woodland savannah and thorn thickets along the Crocodile River, west of Crocodile Bridge, during the dry season.

**Figure 1b.**
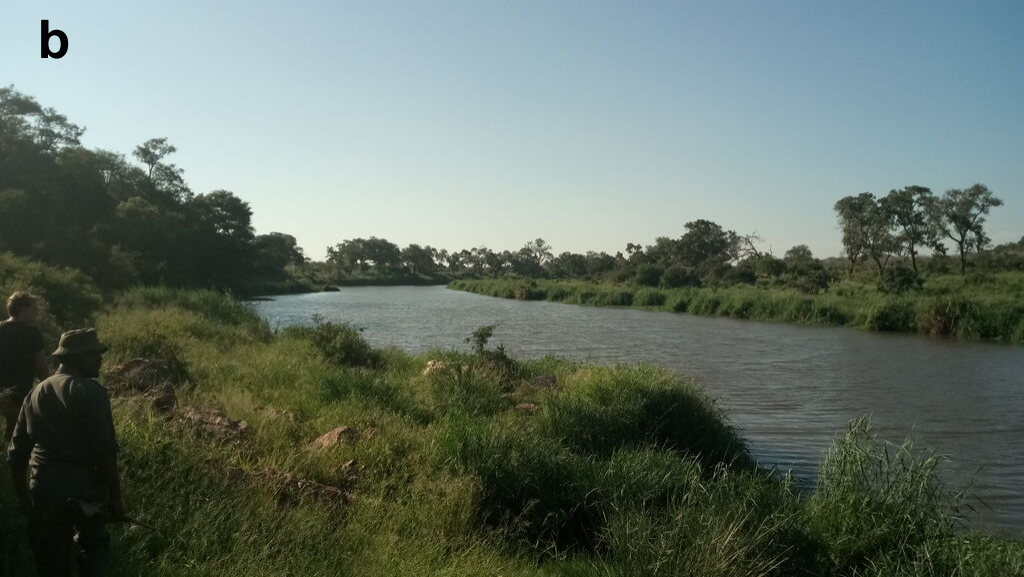
Mixed woodland savannah and thorn thickets along the Crocodile River, west of Crocodile Bridge, during the wet season.

**Figure 1c.**
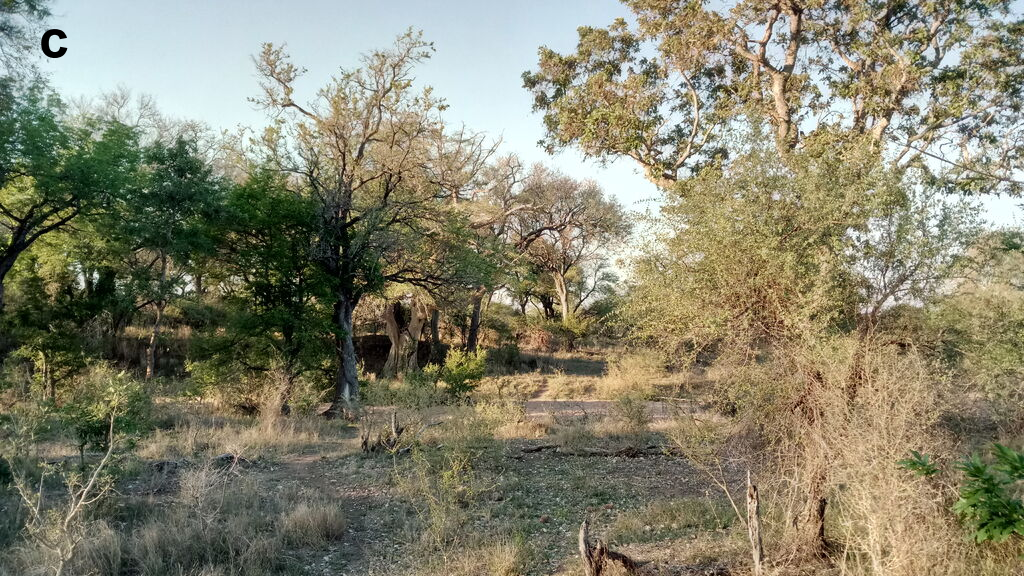
Mixed thornveld and woodland savannah along a seasonal river in the Olifants rugged veld, east of Satara.

**Figure 1d.**
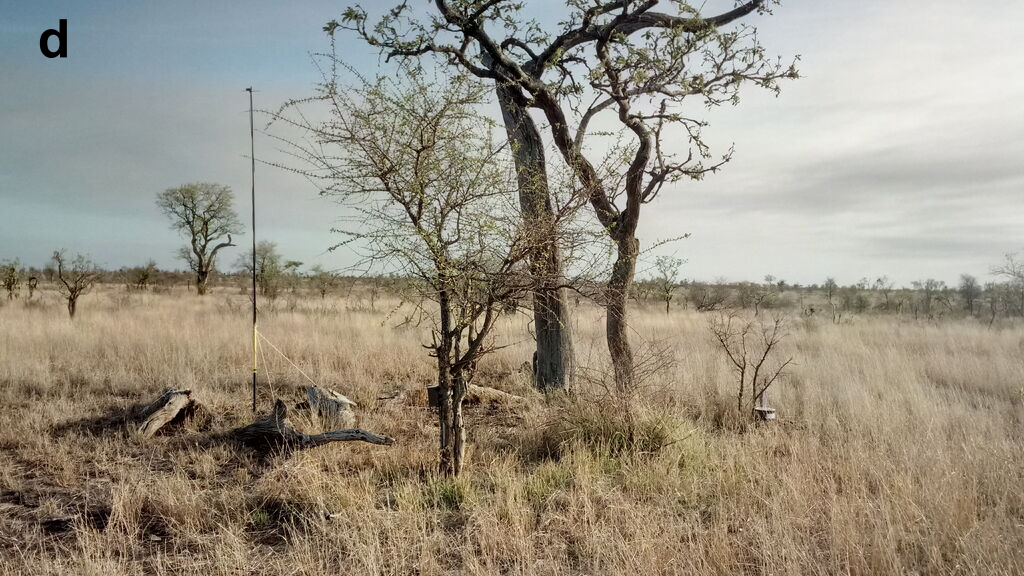
Open savannah grassland on a basalt crest, southeast of Satara.

**Figure 1e.**
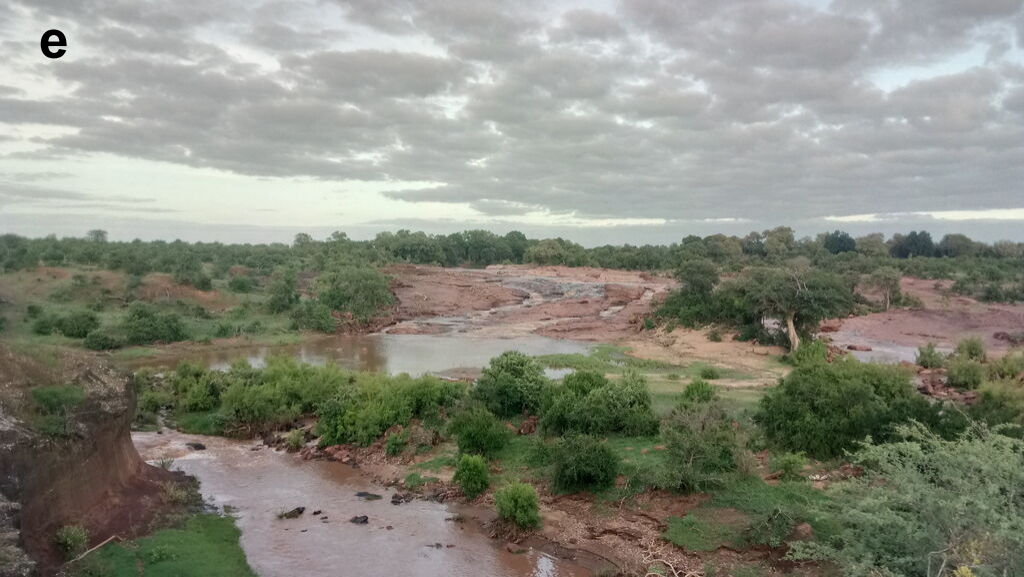
Mopani dominated open savannah along the Shingwedzi River, Red Rocks, west of the Shingwedzi Camp.

**Figure 1f.**
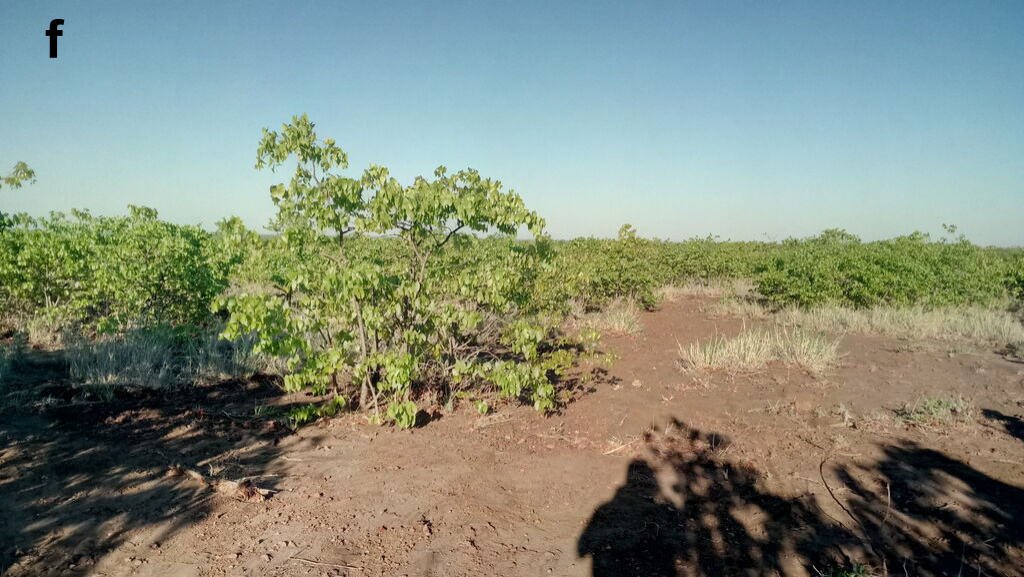
Mopani dominated mosaic of woodland and open savannah on a crest, close to the Shingwedzi tourist Camp.
